# Safety and Efficacy of Vadadustat Once Daily and Three Times Weekly in Patients With Dialysis-Dependent CKD With Anemia

**DOI:** 10.34067/KID.0000000567

**Published:** 2024-09-04

**Authors:** Laura Kooienga, Steven Burke, Amarnath Kathresal, Wenli Luo, Zhihui Yang, Zhiqun Zhang, Rafal Zwiech, German T. Hernandez

**Affiliations:** 1 Colorado Kidney Care, Aurora, Colorado; 2 Akebia Therapeutics, Inc., Cambridge, Massachusetts; 3 Durham Nephrology Associates, PA, Durham, North Carolina; 4 Norbert Barlicki Memorial Teaching Hospital, Lodz, Poland; 5 El Paso Kidney Specialists, El Paso, Texas

**Keywords:** anemia, chronic dialysis, chronic hemodialysis, CKD, dialysis, hemodialysis, hypoxia, kidney disease, renal dialysis

## Abstract

**Key Points:**

- In this phase 3b, noninferiority trial, vadadustat once daily was noninferior to darbepoetin alfa (DA) in the correction and maintenance of hemoglobin in dialysis-dependent CKD.
- Vadadustat three times weekly treatment resulted in similar changes in mean hemoglobin levels compared with vadadustat once daily, but was not noninferior to DA.
- The safety profiles of vadadustat once daily and vadadustat three times weekly were comparable with that of DA.

**Background:**

Vadadustat is an oral hypoxia-inducible factor prolyl hydroxylase inhibitor for treating anemia in CKD. This study investigated the safety and efficacy of once-daily and three-times-weekly dosing in patients with dialysis-dependent CKD compared with darbepoetin alfa (DA).

**Methods:**

This phase 3b, randomized (1:1:1; vadadustat once daily [starting dose: 300 or 450 mg], vadadustat three times weekly [starting dose: 600 or 750 mg], DA), open-label, active-controlled, noninferiority trial included conversion (weeks 0–20) and maintenance (weeks 20–52) periods. Primary and secondary efficacy end points were mean change in hemoglobin from baseline during the primary evaluation period (PEP, weeks 20–26) and secondary evaluation period (weeks 46–52). Other end points included proportion of patients requiring erythropoiesis-stimulating agent (ESA) rescue (hemoglobin <9.5 g/dl or with increases in dose ≥50% or ≥100% in the DA group). Safety end points included treatment-emergent adverse events (AEs) and serious AEs.

**Results:**

The least-squares (LS) mean treatment difference between vadadustat once daily and DA from baseline to PEP was −0.27 g/dl (95% confidence interval [CI], −0.55 to 0.01); the lower bound met the noninferiority threshold (−0.75 g/dl). The LS mean treatment difference between vadadustat three times weekly and DA from baseline to PEP was −0.53 g/dl (95% CI, −0.80 to −0.25), which did not meet the lower bound noninferiority threshold. The LS mean change from baseline to the secondary evaluation period between DA and vadadustat once daily was −0.40 (95% CI, −0.79 to −0.02) and for vadadustat three times weekly was −0.42 (95% CI, −0.81 to −0.02). The proportion of patients who received ESA rescue during weeks 2–52 was higher in the DA group than vadadustat groups. Similar treatment-emergent AEs and treatment-emergent serious AEs were observed across groups.

**Conclusions:**

Vadadustat once daily, but not three times weekly, was noninferior to DA in the correction and maintenance of hemoglobin in patients with dialysis-dependent CKD converted from an ESA; safety profiles were similar across groups.

**Clinical Trial registry name and registration number:**

EudraCT 2019-004851-36/ClinicalTrials.gov identifier: NCT04313153.

## Visual Abstract

**Figure:**
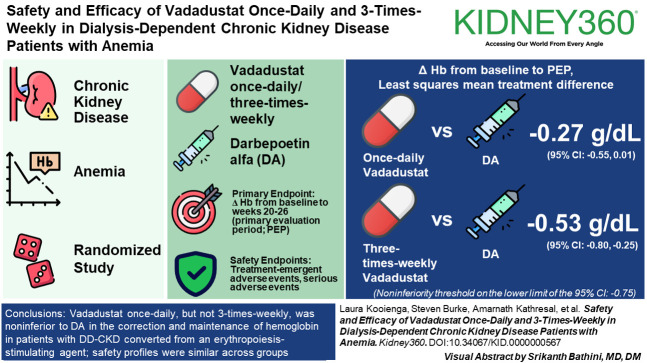

## Introduction

Anemia is a common complication of CKD that largely results from low erythropoietin production because of declining kidney function.^1^ Anemia prevalence increases with CKD progression and is associated with diminished health-related quality of life,^2^ excess health care costs, cardiovascular events, and mortality.^3–6^

Current treatment options for anemia in CKD include iron supplementation, erythropoiesis-stimulating agents (ESAs), and red blood cell (RBC) transfusions.^7–9^ Targeting normal or near-normal hemoglobin levels with ESAs is linked to increased cardiovascular morbidity and mortality; all major guidelines advise caution in ESA use.^7,9,10^ Parenteral ESA administration is labor intensive and requires refrigeration; thus, exploring alternative oral treatments for anemia in patients with dialysis-dependent (DD) CKD may be beneficial.^11,12^

Hypoxia-inducible factor prolyl hydroxylase inhibitors (HIF-PHIs) provide an oral treatment option in patients with anemia associated with CKD.^13–15^ HIF-PHIs stabilize hypoxia-inducible factor, a transcription factor that regulates hypoxia-sensitive genes, resulting in increased endogenous erythropoietin production, improved iron availability, and RBC production.^16–19^ Vadadustat, an oral HIF-PHI, is currently approved for treating anemia in patients with CKD in Japan, in patients with DD-CKD in Europe, and in patients with DD-CKD who have been receiving dialysis for at least 3 months in the United States.^20–22^

In the global phase 3 INNO_2_VATE trials, which included 3923 patients with DD-CKD, vadadustat demonstrated noninferiority to darbepoetin alfa (DA) for the primary safety end point of time to first major adverse cardiovascular event (MACE; defined as all-cause mortality, nonfatal myocardial infarction, or stroke) and the primary efficacy end point of correcting and maintaining hemoglobin levels across region-specific target levels. In addition, the overall safety profile of vadadustat was comparable with DA in DD-CKD populations.^14^

In the INNO_2_VATE trials, vadadustat was initiated at a dose of 300 mg once daily.^14^ Some patients switching from ESAs to vadadustat, particularly those receiving high-dose ESAs at baseline, experienced an initial decline in hemoglobin that resolved with an increased dose. This trial evaluates the efficacy and safety of a higher once-daily starting dose of vadadustat in patients on a high baseline ESA dose, with titration at 2 weeks in those with a declining hemoglobin level. In addition, because patients undergoing in-center three-times-weekly hemodialysis often receive ESAs concomitantly with a dialysis session,^23,24^ we explored a three-times-weekly vadadustat dosing regimen that aligns with the typical dialysis schedule.

## Methods

### Trial Design

We conducted a phase 3b, randomized, open-label, active-controlled, sponsor-blinded trial to assess the efficacy and safety of oral vadadustat both once daily and three times weekly versus DA for the maintenance treatment of anemia in patients with DD-CKD after conversion from ESA therapy (MO_2_DIFY trial; ClinicalTrials.gov identifier: NCT04313153 [date of registration March 18, 2020]; EudraCT 2019-004851-36). The trial was conducted in accordance with the International Conference for Harmonisation guidelines, Good Clinical Practice standards, and relevant local regulatory requirements and laws. The principles outlined in the Declaration of Helsinki were followed. Institutional Review Board approval was obtained from each participating center, and all patients provided written informed consent before enrollment.

### Trial Population

Patients eligible for the trial were age ≥18 years and receiving chronic outpatient in-center hemodialysis three times weekly for ESKD for a minimum of 12 weeks before screening. Patients were required to have used an approved ESA for at least 8 weeks before screening and have had two hemoglobin values at least 4 days apart within the following geographic-specific ranges: the United States: 8–11 g/dl; Europe: 9–12 g/dl. Patients were required to have a serum ferritin concentration ≥100 ng/ml and a transferrin saturation (TSAT) ≥20%. Patients were excluded if the investigator judged anemia to be caused by factors other than CKD or if they had uncontrolled hypertension or recent cardiovascular events. Patients on mean weekly doses of methoxy polyethylene glycol-epoetin beta >50 *μ*g/wk, DA >100 *μ*g/wk, or epoetin alfa analogs >23,000 IU/wk were also excluded. A full list of inclusion and exclusion criteria is provided in Supplemental Table 1.

### Trial Procedures

After a screening period of up to 8 weeks (56 days), patients were randomized 1:1:1 to receive vadadustat once daily, vadadustat three times weekly, or DA, and were stratified by geographic region (the United States/Europe) and mean baseline weekly DA dose (or ESA equivalent) calculated over a period of 8 weeks before screening visit 2 (*i.e*., low ESA-dose group [≤0.45 *μ*g/kg per week]; high ESA-dose group [>0.45 and ≤1.5 *μ*g/kg per week]). After randomization and screening, the trial had two defined phases: a conversion and maintenance period (weeks 0–52), which included conversion to the investigational product (weeks 0–20), the prespecified primary evaluation period (PEP; weeks 20–26) and secondary evaluation period (SEP; weeks 46–52), and a safety follow-up period of 4 weeks.

Vadadustat was provided in oral tablets with dosages of 150 and 450 mg. For the low ESA-dose group, the starting vadadustat doses were either 300 mg once daily or 600 mg three times weekly. The starting vadadustat dose for the high ESA-dose group was either 450 mg once daily or 750 mg three times weekly. The minimum vadadustat dose was 150 mg, and the maximum dose could be adjusted up to 900 mg once daily or 1200 mg three times weekly. Both vadadustat and DA doses were titrated using protocol-specified algorithms to maintain target hemoglobin levels (10–11 g/dl in the United States; 10–12 g/dl in Europe). Iron supplementation was permitted and recommended to sustain serum ferritin concentrations at or above 100 ng/ml and TSAT at or above 20%. For more details on patient dosing, please see the Supplemental Methods.

Patients in all treatment groups were eligible to receive an ESA or RBC transfusion as rescue therapy if they experienced worsening symptoms of anemia, with hemoglobin concentration <9.5 g/dl. Rescue therapy was halted at hemoglobin levels ≥10.0 g/dl, following the local institution's guidelines and the product label. During rescue therapy, vadadustat was temporarily discontinued while patients received an ESA. RBC transfusion was administered as clinically indicated, and trial drugs were continued. Details on treatment rescue for anemia are included in the Supplemental Methods.

### End Points

The primary efficacy end point assessed the mean change in hemoglobin concentration from baseline to PEP. The key secondary efficacy end point assessed the change in hemoglobin levels between baseline and SEP. Subgroup analyses of mean change in hemoglobin concentration were completed by DA baseline dose (low [≤0.45 *μ*g/kg per week] versus high [>0.45 and ≤1.5 *μ*g/kg per week]) and by region with corresponding hemoglobin targets (the United States: 10–11 g/dl; Europe: 10–12 g/dl).

The prespecified safety end points included the incidence of adverse events (AEs), serious AEs (SAEs), treatment-emergent AEs (TEAEs), treatment-emergent SAEs, and AEs of special interest (AESIs; events included as AESIs are detailed in the Supplemental Methods), laboratory assessments, hemoglobin excursions, and hemoglobin rate of rise. Deaths and any suspected nonfatal myocardial infarctions, strokes, hospitalizations for heart failure (HF), or major thromboembolic events were adjudicated in a blinded fashion by a Safety Event Adjudication Committee. A full list of efficacy and safety end points is included in Supplemental Table 2. Iron indices were assessed as pharmacodynamic markers (total iron-binding capacity, serum iron, ferritin, and TSAT collected at baseline and every 4 weeks throughout the trial.

### Statistical Analyses

The data presented here are from the randomized population, the full analysis set, the per-protocol population, and the safety population (full details on analysis populations are provided in the Supplemental Methods). For efficacy, the randomized population was analyzed using analysis of covariance with stratification factors and baseline hemoglobin as covariates. Noninferiority of vadadustat was determined if the lower limit of the 95% confidence interval (CI) for the difference in mean hemoglobin change between vadadustat and DA was above −0.75 g/dl (full details on determination of noninferiority are provided in the Supplemental Methods). A hierarchical testing scheme corrected for multiple comparisons between vadadustat once daily versus DA and vadadustat three times weekly versus DA.

Data from the safety population were analyzed using descriptive statistics to summarize continuous variables and to tabulate categorical variables by frequency count and percentage. Counts, proportions, events, and event rates per 100 patient-years were calculated for patients who experienced AESIs. Odds ratios and 95% CIs were also calculated to compare treatment arms. Further details on data imputation are provided in the Supplemental Methods.

## Results

### Patient Demographics, Baseline Characteristics, and Disposition

A total of 319 patients were randomized, with two patients not treated because they were considered screen failures, and the remaining patients were assigned to treatment with vadadustat once daily (*n*=105), vadadustat three times weekly (*n*=104) or DA (*n*=108) (Supplemental Figure 1). The safety population consisted of 317 patients, and the full analysis set and per-protocol populations consisted of 313 and 190 patients, respectively. Overall, demographics and baseline characteristics were well balanced between treatment groups in the randomized (Table 1) and per-protocol populations (Supplemental Table 3).

**Table 1:** Selected demographic baseline characteristics (randomized population)

Characteristic	Vadadustat Once Daily (*n*=105)	Vadadustat Three Times Weekly (*n*=106)	DA (*n*=108)
Mean age, yr (SD)	60.9 (13.4)	61.2 (12.5)	60.8 (12.8)
Sex, male, *No.* (%)	58 (55.2)	60 (56.6)	65 (60.2)
**Racial or ethnic group, *No.* (%)**			
Asian	4 (3.8)	1 (0.9)	3 (2.8)
Black	31 (29.5)	30 (28.3)	33 (30.6)
Other^a^	2 (2.0)	8 (7.5)	1 (0.9)
White	68 (64.8)	67 (63.2)	71 (65.7)
**Hispanic ethnic group, *No.* (%)**			
Hispanic/Latino	23 (21.9)	36 (34.0)	26 (24.1)
Not Hispanic/Latino	82 (78.1)	70 (66.0)	82 (75.9)
**Region of enrollment, *No.* (%)**			
The United States	75 (71.4)	77 (72.6)	77 (71.3)
Europe	30 (28.6)	29 (27.4)	31 (28.7)
Dry weight, kg, mean (SD)	83.3 (22.1)	82.3 (20.8)	85.8 (20.8)
Mean BMI, kg/m^2^ (SD)	29.4 (7.3)	29.1 (6.6)	29.7 (6.2)
**Baseline ESA use, *No.* (%)**			
Epoetin alfa	52 (49.5)	42 (39.6)	44 (40.7)
DA	19 (18.1)	23 (21.7)	31 (28.7)
Methoxy polyethylene glycol-epoetin beta	34 (32.4)	41 (38.7)	33 (30.6)
**DA (ESA equivalent dose), *μ*g/kg per week, *No.* (%)**			
≤0.45	80 (76.2)	84 (79.2)	85 (78.7)
>0.45 and ≤1.5	25 (23.8)	21 (19.8)	23 (21.3)
**Baseline ESA dose, U/kg per week**			
Mean (SD)	65.8 (56.4)	68.3 (55.2)	64.0 (47.1)
Median (Q1–Q3)	46.0 (24.0–78.0)	52.0 (26.0–84.0)	54.0 (26.0–85.0)
≤90	83 (79.0)	84 (79.2)	84 (77.8)
>90 and <300	22 (21.0)	22 (20.8)	24 (22.2)
**Baseline Hb**			
Concentration, g/dl, mean (SD)	10.3 (0.71)	10.2 (0.74)	10.2 (0.81)
*<10 g/dl, No. (%)*	34 (32.4)	39 (36.8)	40 (37.0)
*≥10 g/dl, No. (%)*	71 (67.6)	67 (63.2)	68 (63.0)
Iron (*μ*g/dl), mean (SD)	81.2 (36.45)	77.4 (30.86)	77.8 (32.87)
Ferritin (ng/ml), mean (SD)	714.8 (336.9)	719.9 (409.1)	663.9 (387.6)
TSAT (%), mean (SD)	38.8 (13.91)	38.8 (13.56)	39.7 (14.03)
C-reactive protein (mg/dl), mean (SD)	0.90 (1.35)	1.13 (3.12)	1.16 (2.21)
**Iron use, *No****.* **(%)**			
Patient not receiving any iron	26 (24.8)	35 (33.0)	31 (28.7)
Patient receiving IV iron only	72 (68.6)	63 (59.4)	68 (63.0)
*IV iron (mg/wk), mean (SD)*	54.7 (33.5)	59.0 (49.5)	68.5 (55.0)
Years since chronic dialysis initiation, mean (SD)	4.4 (3.9)	4.2 (4.2)	3.9 (3.4)
**Dialysis adequacy^b^**			
Kt/V, mean (SD)	1.6 (0.3)	1.6 (0.3)	1.7 (0.5)
**Vascular access type^b^, *No.* (%)**			
AVF	73 (69.5)	70 (67.3)	88 (81.5)
AVG	20 (19.0)	15 (14.4)	8 (7.4)
Temporary catheter	1 (1.0)	2 (1.9)	2 (1.9)
Tunneled dialysis catheter	10 (9.5)	15 (14.4)	8 (7.4)
Other	0 (0.0)	0 (0.0)	2 (1.9)
**NYHA CHF class, *No.* (%)**			
0 or 1	89 (84.8)	83 (78.3)	77 (71.3)
2 or 3	10 (9.5)	13 (12.3)	20 (18.5)
Unknown	6 (5.7)	10 (9.4)	11 (10.2)
**Comorbidities, *No.* (%)**			
Diabetes	66 (62.9)	69 (65.1)	74 (68.5)
Cardiovascular disease (coronary artery disease, myocardial infarction, stroke, and HF)	48 (45.7)	45 (42.5)	52 (48.1)
Other cardiovascular disease (deep venous thrombosis, arterial thrombosis, pulmonary embolism, and vascular access thrombosis)	16 (15.2)	15 (14.2)	10 (9.3)
Hyperparathyroidism			
*Primary*	0 (0.0)	1 (0.9)	1 (0.9)
*Secondary*	91 (86.7)	95 (89.6)	96 (88.9)

AVF, arteriovenous fistula; AVG, arteriovenous graft; BMI, body mass index; DA, darbepoetin alfa; ESA, erythropoiesis-stimulating agent; Hb, hemoglobin; HF, heart failure; IV, intravenous; Kt/V, clearance, time and volume (ratio of the amount of solute removed (clearance times time) to the volume of distribution of that solute in the body); NYHA CHF, New York Heart Association congestive heart failure; TSAT, transferrin saturation.

a Includes American Indian or Alaska Native, Native Hawaiian or other Pacific Islander, multiple, any other, or not reported.

b Vadadustat three times weekly, *n*=104.

### Primary, Key Secondary, and Other Efficacy End Points

#### Primary Efficacy End Point: Randomized Population

**Figure 1:**
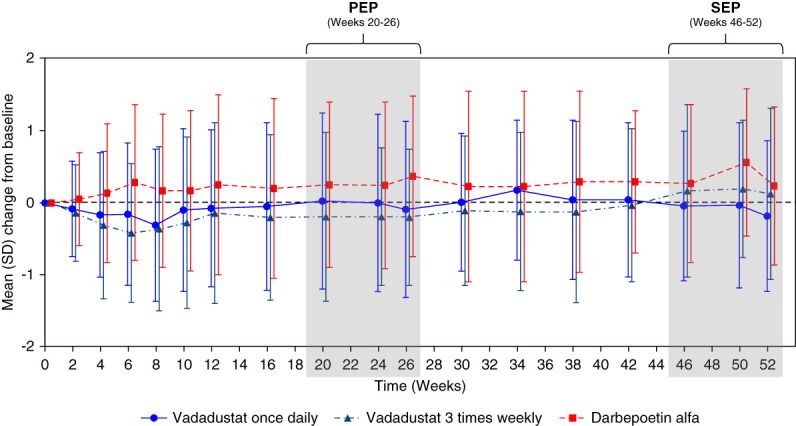
**Mean change in hemoglobin from baseline over time.** PEP, primary evaluation period; SEP, secondary evaluation period.

Throughout the PEP and SEP, hemoglobin levels remained consistent in the vadadustat once-daily group relative to baseline, higher than baseline in the DA group, and lower than baseline for the vadadustat three-times-weekly group (Figure 1 and Supplemental Figure 2). The vadadustat once daily group was noninferior to DA during the PEP (least-squares [LS] mean treatment difference: –0.27 g/dl; 95% CI, –0.55 to 0.01), with the lower limit of the 95% CI above the prespecified noninferiority margin of –0.75 g/dl. However, the LS mean treatment difference between the vadadustat three times weekly and DA groups did not meet the threshold of noninferiority (–0.53 g/dl; 95% CI, –0.80 to –0.25) (Table 2 and Supplemental Figure 3).

**Table 2:** Change in hemoglobin from baseline during primary and secondary evaluation periods (randomized and per protocol populations)

Characteristic	Vadadustat Once Daily	Vadadustat Three Times Weekly	DA
Randomized population, *No.*	105	106	108
**PEP (weeks 20-26)**			
Baseline Hb concentration, g/dl, mean (SD)	10.30 (0.71)	10.19 (0.74)	10.19 (0.81)
Hb concentration, g/dl, mean (SD)	10.22 (1.13)	9.94 (0.96)	10.47 (0.91)
Change in Hb from baseline,^a^ LS mean (95% CI)	0.07 (–0.17 to 0.31)	–0.19 (–0.41 to 0.04)	0.34 (0.11 to 0.57)
Difference in Hb change from DA, LS mean (95% CI)	–0.27 (–0.55 to 0.01)^b^	–0.53 (–0.80 to –0.25)	NA
**SEP (weeks 46–52)**			
Hb concentration, g/dl, mean (SD)	10.20 (1.00)	10.17 (1.32)	10.59 (1.16)
Change in Hb from baseline,^a^ LS mean (95% CI)	0.04 (–0.26 to 0.34)	0.03 (–0.28 to 0.33)	0.44 (0.14 to 0.75)
Difference in Hb change from DA, LS mean (95% CI)	–0.40 (–0.79 to –0.02)	–0.42 (–0.81 to –0.02)	NA
Per-protocol population, *No.*	62	64	64
**PEP (weeks 20–26)**			
Baseline Hb concentration, g/dl, mean (SD)	10.30 (0.69)	10.28 (0.77)	10.26 (0.68)
Hb concentration, g/dl, mean (SD)	10.35 (1.01)	10.05 (0.89)	10.50 (0.81)
Change in Hb from baseline,^a^ LS mean (95% CI)	0.18 (−0.07 to 0.43)	–0.17 (–0.40 to 0.05)	0.27 (0.04 to 0.49)
Difference in Hb change from DA, LS mean (95% CI)	–0.09 (–0.38 to 0.21)^b^	–0.44 (–0.73 to –0.16)^b^	NA
**SEP (weeks 46–52)**			
Hb concentration, g/dl, mean (SD)	10.31 (0.88)	10.36 (1.28)	10.72 (1.01)
Change in Hb from baseline,^a^ LS mean (95% CI)	0.06 (–0.28 to 0.40)	0.07 (–0.23 to 0.38)	0.43 (0.15 to 0.72)
Difference in Hb change from DA, LS mean (95% CI)	–0.37 (–0.76 to 0.01)	–0.36 (–0.75 to 0.02)^b^	NA

CI, confidence interval; DA, darbepoetin alfa; Hb, hemoglobin; LS, least-squares; NA, not applicable; PEP, primary evaluation period; SEP, secondary evaluation period.

a Derived from an analysis of covariance with randomization stratification factors and baseline hemoglobin as covariates.

b Above prespecified noninferiority margin: lower bound of the 95% CI of difference in change in hemoglobin ≥−0.75 g/dl.

#### Primary Efficacy End Point: Per-Protocol Population

Both vadadustat once daily and vadadustat three times weekly demonstrated noninferiority to DA for change in hemoglobin concentrations from baseline in the per-protocol population during the PEP. The LS mean treatment difference of the vadadustat once-daily group compared with DA was –0.09 g/dl (95% CI, –0.38 to 0.21) with the lower bound of the CI within the noninferiority margin. In the vadadustat three-times-weekly group compared with the DA group, the LS mean treatment difference was –0.44 g/dl (95% CI, –0.73 to –0.16), also surpassing the noninferiority margin (Table 2 and Supplemental Figure 2).

#### Secondary Efficacy End Points

For the randomized population, during the SEP, the LS mean treatment difference in hemoglobin concentration between DA and vadadustat once daily was –0.40 g/dl (95% CI, –0.79 to –0.02) (Table 2 and Supplemental Figure 2). The LS mean treatment difference between DA and vadadustat three times weekly was –0.42 g/dl (95% CI, –0.81 to –0.02). The lower limit of the 95% CI exceeded the prespecified noninferiority margin of –0.75 g/dl in both cases. The results for the secondary efficacy end points were similar in the per-protocol population analyses.

Mean hemoglobin concentrations remained within target ranges specified by region throughout the PEP. The proportion of patients in the randomized population with an average hemoglobin value within the target range was 51.0% (95% CI, 47.6 to 54.3) in the vadadustat once-daily group, 50.7% (95% CI, 47.2 to 53.8) in the vadadustat three-times-weekly group, and 54.5% (95% CI, 50.9 to 58.3) in the DA group. The odds ratio treatment comparison between vadadustat once daily and DA was 0.87 (95% CI, 0.48 to 1.59), similar to the treatment comparison between vadadustat three times weekly and DA (0.86 [95% CI, 0.47 to 1.60]). Similar results were observed in the SEP (Supplemental Table 4).

#### Subgroup Analysis of Change in Hemoglobin by Region

At baseline, US patients had lower mean hemoglobin concentrations compared with patients in Europe (Supplemental Table 5). The change in LS mean hemoglobin concentrations was 0.31 g/dl (95% CI, 0.07 to 0.55) and 0.33 g/dl (95% CI, –0.02 to 0.68) during the PEP and SEP, respectively, among US patients in the DA treatment group. In European patients treated with DA, mean hemoglobin concentrations increased marginally during the SEP (0.22 g/dl [95% CI, –0.33 to 0.77]).

#### Subgroup Analysis of Change in Hemoglobin by Baseline ESA Dose

In patients treated with vadadustat three times weekly, mean hemoglobin concentrations were consistently lower than baseline through the PEP (Figure 1 and Table 2). Subgroup analyses in the low baseline DA dose (≤0.45 *μ*g/kg per week) subgroup, which had a starting dose of 600-mg vadadustat, showed the LS mean change in hemoglobin from baseline to PEP was –0.28 (95% CI, –0.50 to –0.06) (Supplemental Table 6). The high baseline DA dose (>0.45 and ≤1.5 *μ*g/kg per week) subgroup, which had a starting dose of 750-mg vadadustat, had an LS mean change from baseline to PEP of 0.24 g/dl (95% CI, –0.32 to 0.80). Similar results were seen in the vadadustat once-daily group in the high and low baseline ESA dose subgroups (Supplemental Table 6).

#### Average Weekly Dose of Study Treatment

Overall, patients in the vadadustat three-times-weekly group received lower mean weekly doses than those in the vadadustat once-daily group (Supplemental Table 7). During the PEP, patients in the vadadustat three-times-weekly group received approximately 26.7% lower mean weekly doses than those in the vadadustat once-daily group (vadadustat once daily: 2945.1 mg; vadadustat three times weekly: 2160.2 mg; difference: 784.9 mg). Similar results were seen during the SEP (Supplemental Table 7).

#### Proportion of Patients Receiving ESA Rescue or RBC Transfusion

In general, the proportion of patients receiving ESA rescue because of hemoglobin <9.5 g/dl was higher in the DA versus the vadadustat groups. In the PEP (weeks 20–26), the proportions of patients who received any ESA rescue (increases of ≥50% in dose within the DA group) were 7.6%, 9.8%, and 15.6% for the vadadustat once-daily, vadadustat three-times-weekly, and DA groups, respectively. The proportions of patients receiving ESA rescue during the SEP (weeks 46–52) were 1.5%, 1.5%, and 12%, respectively. Similar results were seen for an increase of ESA dose by >100% in PEP and SEP (Supplemental Table 8).

During the entire study period, the proportions of randomized patients receiving any RBC transfusions were 1.0%, 3.9%, and 1.9% in the vadadustat once-daily, vadadustat three-times-weekly, and DA groups, respectively (Supplemental Table 9).

### Safety End Points

#### Summary of AEs

Drug-related TEAEs were observed in 12.4% and 16.3% of patients in the vadadustat once-daily and three-times-weekly groups, respectively, and included gastrointestinal disorders (diarrhea [1.9%], nausea [1.9%], and vomiting [1.9%] in the vadadustat once-daily group; diarrhea [7.7%] and abdominal pain [1.9%] in the vadadustat three-times-weekly group). No drug-related TEAEs were reported in the DA group.

The proportions of patients with TEAEs leading to study drug discontinuation were 2.9%, 10.6%, and 2.8% in the vadadustat once-daily, vadadustat three-times-weekly, and DA groups, respectively (Table 3). Most discontinuations were associated with gastrointestinal disorders.

**Table 3:** Overall summary of adverse events (safety population)

Event	Vadadustat Once Daily (*n*=105)	Vadadustat Three Times Weekly (*n*=104)	DA (*n*=108)
	*No*. (%)	*No*. (%)	*No*. (%)
Any TEAEs	89 (84.8)	88 (84.6)	87 (80.6)
Any drug-related TEAEs	13 (12.4)	17 (16.3)	0 (0.0)
Any severe TEAEs	27 (25.7)	35 (33.7)	32 (29.6)
Any serious TEAEs	47 (44.8)	47 (45.2)	47 (43.5)
Any drug-related serious TEAEs	1 (1.0)	1 (1.0)	0 (0.0)
Any TEAEs leading to study drug discontinuation	3 (2.9)	11 (10.6)	3 (2.8)
Any drug-related TEAEs leading to study drug discontinuation	3 (2.9)	5 (4.8)	0 (0.0)
Any TEAEs resulting in death	12 (11.4)	9 (8.7)	7 (6.5)
Any deaths	12 (11.4)	9 (8.7)	7 (6.5)

DA, darbepoetin alfa; TEAEs, treatment-emergent adverse events.

The most frequently (≥2%) reported SAEs by system-organ class for the treatment groups (vadadustat once-daily, vadadustat three-times-weekly, and DA) were infections and infestations (18.1%, 21.2%, and 19.4%); cardiac disorders (9.5%, 13.5%, and 7.4%); injury, poisoning, and procedural complications (7.6%, 8.7%, and 6.5%); metabolism and nutrition disorders (6.7%, 6.7%, and 8.3%); and blood and lymphatic system disorders (3.8%, 6.7%, and 4.6%), respectively.

One drug-related treatment-emergent SAE (vadadustat once-daily group: elevated alanine aminotransferase [ALT] and aspartate aminotransferase [AST] >3 times the upper limit of normal [ULN]; vadadustat three-times-weekly group: hypoglycemia) was reported in each vadadustat group (Table 3 and Supplemental Table 10). None were observed in the DA group.

TEAEs resulting in death were reported in 12 (11.4%), 9 (8.7%), and 7 (6.5%) patients in the vadadustat once-daily, vadadustat three-times-weekly, and DA groups, respectively. No deaths were considered study drug related. The leading cause of death in all groups was coronavirus disease 2019 (COVID-19) pneumonia.

#### AESIs

The frequency of any AESIs was 27.6% in the vadadustat once-daily group, 38.5% in the vadadustat three-times-weekly group, and 31.5% in the DA group (Supplemental Table 11). Worsening of hypertension was experienced by 6.7%, 13.5%, and 10.2% of patients in the vadadustat once-daily, vadadustat three-times-weekly, and DA groups, respectively. Hepatotoxicity TEAEs, including narrow Standardised MedDRA Query drug-related hepatic disorders comprehensive, were observed in 3.8%, 2.9%, and 4.6% of patients in the vadadustat once-daily, vadadustat three-times-weekly, and DA groups, respectively. Pulmonary hypertension TEAEs (new-onset or exaggerated pulmonary hypertension), including narrow Standardised MedDRA Query pulmonary hypertension, were observed in 5.7%, 3.8%, and 2.8% of patients in the vadadustat once-daily, vadadustat three-times-weekly, and DA groups, respectively.

Although infrequent, there was a slightly higher incidence of malignancies, including renal cell carcinoma, in the DA group (3.7%) than in the vadadustat once-daily (1.0%) or vadadustat three-times-weekly (2.9%) groups. The incidence of thrombosis was similar in the vadadustat once-daily (10.5%) group, vadadustat three-times-weekly (14.4%) group, and DA (12.0%) group. Device/shunt thrombosis/occlusion/malfunction/stenosis occurred more frequently in the DA group (12%) than in the vadadustat once-daily (5.7%) and vadadustat three-times-weekly (9.6%) groups. There was a higher incidence of congestive HF in the vadadustat once-daily (8.6%, *n*=9) and vadadustat three-times-weekly (6.7%, *n*=7) groups than in the DA group (1.9%, *n*=2). None of the events were considered drug related. Eight patients in the vadadustat groups and one patient in the DA group had a history of HF. Five patients in the vadadustat groups and no patients in DA group had hospitalization for HF with concomitant infection with COVID-19 or non-COVID pneumonia. The incidence of stroke and sepsis/septic shock was lower in the vadadustat once-daily group (1.9%) than in the vadadustat three-times-weekly (4.8%) and DA (4.6%) groups.

#### Adjudicated Safety Events

The proportions of patients with any MACE were 13.3%, 13.4%, and 9.2%, and with MACE plus hospitalizations for HF or thromboembolic events (excluding vascular access thrombosis) were 17.1%, 18.2%, and 11.1% in the vadadustat once-daily, vadadustat three-times-weekly, and DA groups, respectively (Table 4). The incidence of an adjudicated HF event was higher in the vadadustat once-daily (6.6%, *n*=7) and vadadustat three-times-weekly (4.8%, *n*=5) groups than in the DA group (1.8%, *n*=2). However, none of these events in the vadadustat groups were drug related. Of patients with an adjudicated HF event, six patients in the vadadustat groups and one patient in the DA group had a history of HF. Two patients had concomitant infections (COVID-19 or non-COVID pneumonia) in the vadadustat groups, with no concomitant infections in the DA group.

**Table 4:** Summary of adjudicated safety events (safety population)

*No.* (%)	Vadadustat Once Daily (*n*=105)		Vadadustat Three Times Weekly (*n*=104)		DA (*n*=108)	
	*No.* (%)	E (E×100/PY)	*No.* (%)	E (E×100/PY)	*No.* (%)	E (E×100/PY)
Any MACE^a^	14 (13.3)	15 (17.3)	14 (13.4)	18 (20.9)	10 (9.2)	10 (10.4)
Any MACE plus hospitalization for HF or thromboembolic event excluding vascular access thrombosis	18 (17.1)	25 (28.8)	19 (18.2)	26 (30.2)	12 (11.1)	12 (12.5)
Any death	12 (11.4)	12 (13.8)	9 (8.6)	9 (10.5)	7 (6.4)	7 (7.3)
Nonfatal stroke	0 (0.0)	0 (0.0)	0 (0.0)	0 (0.0)	2 (1.8)	2 (2.1)
Myocardial infarction	2 (1.9)	3 (3.5)	7 (6.7)	9 (10.5)	1 (0.9)	1 (1.0)
Thromboembolic event	3 (2.8)	3 (3.5)	3 (2.8)	3 (3.5)	2 (1.8)	2 (2.1)
Hospitalization for HF	7 (6.6)	9 (10.4)	5 (4.8)	5 (5.8)	2 (1.8)	2 (2.1)

DA, darbepoetin alfa; E, events; HF, heart failure; MACE, major adverse cardiovascular event; PY, patient-years.

a Any MACE: all-cause mortality, nonfatal myocardial infarction, nonfatal stroke.

#### Liver Safety

The incidence of liver function test abnormalities was comparable across treatment groups (Supplemental Table 10). In the vadadustat once-daily, one patient experienced increased ALT and AST levels between three and five times the ULN. However, the total bilirubin remained within the normal range. The SAE of increased liver function test was considered unrelated to the study drug. Two patients in the DA group had ALT levels between three and five times the ULN and concomitant total bilirubin levels between two and three times the ULN. However, these SAEs were considered unrelated to the study drug.

#### Hemoglobin-Related Safety End Points

Patients receiving vadadustat once daily and three times weekly had fewer hemoglobin excursions of >11.0, >12.0, and >13.0 g/dl, compared with patients receiving DA at any postbaseline hemoglobin assessment (Supplemental Table 12). Overall, a lower percentage of patients in the vadadustat once-daily (24.8%) and three-times-weekly (19.6%) groups had a mean hemoglobin increase >1.0 g/dl within a 2-week interval compared with the DA group (33.0%) (Supplemental Table 12). A greater proportion of patients with hemoglobin decreases to <9.0 and <8.0 g/dl were reported in the vadadustat three-times-weekly group than the vadadustat once-daily and DA groups.

#### Iron-Related Parameters

Iron-related parameters demonstrated a pharmacodynamically expected increase in total iron-binding capacity and decrease in hepcidin with the initiation of vadadustat regardless of baseline starting dose (Supplemental Table 13).

## Discussion

The present phase 3b clinical study evaluated the efficacy and safety of oral vadadustat once daily and three times weekly compared with DA for the treatment of anemia in patients with DD-CKD after conversion from their prior ESA therapy. In the randomized population, vadadustat once daily demonstrated noninferiority to DA for the primary efficacy end point (change in mean hemoglobin from baseline to weeks 20–26), but vadadustat three times weekly did not. However, in the per-protocol population, vadadustat once daily and three times weekly both demonstrated noninferiority to DA. The present trial used a hierarchical testing procedure to manage comparisons between treatment groups and ensure a low rate of type 1 error (false positives). No claims of noninferiority for the key secondary efficacy end point in either of the vadadustat dose groups versus DA were made. Patients receiving vadadustat once daily and three times weekly both had a lower risk of ESA rescue than those on DA. RBC transfusions were received infrequently across the trial, and rates were comparable across all three treatment groups.

Patients in the vadadustat three-times-weekly group received substantially lower mean weekly doses than those in the vadadustat once-daily group, likely because of protocol design, which limited the three-times-weekly dose to a total of 3600 mg weekly versus 6300 mg for once-daily dosing. During the PEP and SEP, the vadadustat three-times-weekly group received approximately 26.7% and 33.1% lower mean weekly doses, respectively, than the vadadustat once-daily group. It is possible that the lower mean weekly dose of vadadustat in the three-times-weekly group influenced the ability of vadadustat to demonstrate noninferiority in the randomized population. Both vadadustat once daily and vadadustat three times weekly maintained mean hemoglobin within the target range while minimizing hemoglobin excursions and rapid rises compared with DA. To further explore appropriate three-times-weekly dosing, an additional study with 600 mg and 900 mg vadadustat starting doses was conducted (NCT04707768), which found that both starting doses were noninferior to epoetin beta pegol; however, the 600 mg dose required an adjustment period.^25^

Overall, the safety profiles of vadadustat once daily and vadadustat three times weekly were similar to that of DA, apart from a higher incidence of gastrointestinal disorders in the vadadustat groups. AE reporting could be influenced by the open-label nature of the trial. The laboratory safety profiles of both the vadadustat once-daily and vadadustat three-times-weekly groups indicated no clinically meaningful changes in hepatic parameters (*i.e*., ALT, AST, alkaline phosphatase, and total bilirubin) compared with the DA group.

Although this study was not designed to assess cardiovascular safety, cardiovascular events were adjudicated by a blinded committee. No MACEs were reported as drug-related SAEs, and there were no adjudicated drug-related discontinuations in any treatment group. There was a higher incidence of hospitalization for HF in the vadadustat groups (6.6% and 4.8% in the once-daily and three-times-weekly groups, respectively) than in the DA group (1.8%). Most patients either had a history of HF or another precipitating risk factor, such as COVID-19 infection or non-COVID pneumonia. In the much larger global vadadustat INNO_2_VATE clinical program, the incidence of the first adjudicated event of HF was 3.9% (76 events) in the vadadustat group and 4.0% (79 events) in the DA group.^14^ In the ASCEND-ND and ASCEND-D trials, daprodustat, another HIF-PHI, had a higher incidence of HF in patients not on dialysis but a similar incidence in patients on dialysis when compared with DA.^26,27^ Further analyses of these trials suggested that cardiovascular risk factors and coronary artery disease were associated with a greater risk of HF in patients treated with daprodustat.^28^

Other once-daily HIF-PHIs have demonstrated efficacy in treating anemia in patients with CKD undergoing hemodialysis^13,26^; three-times-weekly dosing regimens of these agents have also demonstrated noninferiority to epoetin alfa in hemoglobin response and were generally well tolerated.^29,30^

This trial was limited by the open-label design and small group sizes. In addition, the trial was conducted over a 2-year period that overlapped with the COVID-19 pandemic. The impact of the pandemic led to a higher frequency of COVID-19–related events (including deaths) being reported across all treatment groups. Further, this study was not designed to assess vadadustat in ESA-hyporesponsive patients because they were excluded from the study.

In conclusion, vadadustat administered once daily to patients with DD-CKD, after conversion from their current ESA therapy based on dose, proved noninferior to DA. Although vadadustat administered three times weekly did not meet the criteria for noninferiority, this dosing regimen proved comparable with vadadustat once daily for changes in mean hemoglobin levels. Overall, the safety profiles of vadadustat once daily and vadadustat three times weekly were comparable with that of DA. Vadadustat offers a convenient oral alternative for the treatment of anemia in patients with DD-CKD, circumventing the limitations of parenteral or subcutaneous routes and providing an important option when iron supplementation and ESA treatment prove inadequate.^31^ The results from this study support the safety and efficacy of vadadustat across a range of doses for managing anemia in patients with DD-CKD.

## Supplementary Material

SUPPLEMENTARY MATERIAL

## Data Availability

Proposal requests for access to original data should be sent to medicalinfo@akebia.com. Deidentified patient-level data will be available 12 months after US and EU approval to qualified researchers with an appropriate research proposal. The research proposal is subject to review by an independent review board with final approval by Akebia Therapeutics, Inc.

## Supplemental Material

Supplemental Methods

Supplemental Table 1. Study inclusion and exclusion criteria.

Supplemental Table 2. Trial end points.

Supplemental Table 3. Selected demographic baseline characteristics (per protocol population).

Supplemental Table 4. Proportion of patients with average hemoglobin values within target range (randomized population).

Supplemental Table 5. Change in hemoglobin from baseline during primary and secondary evaluation periods by geographic region (randomized populations).

Supplemental Table 6. Change in hemoglobin from baseline during primary and secondary evaluation periods by baseline vadadustat dose group (randomized populations.

Supplemental Table 7. Average weekly dose of study treatment (safety population).

Supplemental Table 8. Proportion of patients with any ESA rescue medications (randomized population).

Supplemental Table 9. Proportion of patients receiving any red blood cell transfusions (randomized population).

Supplemental Table 10. Abnormal laboratory results related to liver enzymes (safety population).

Supplemental Table 11. Adverse events of special interest (safety population).

Supplemental Table 12. Hemoglobin-related safety end points (safety population).

Supplemental Table 13. Change in iron parameters over the treatment period (safety population).

Supplemental Figure 1. CONSORT flow chart for the MO2DIFY trial.

Supplemental Figure 2. Box and whisker plot of change in hemoglobin over time (randomized population).

Supplemental Figure 3. Change in hemoglobin from baseline to primary and secondary evaluation periods (randomized population).
