# Supplementary material for: Safety and Efficacy of Vadadustat Once Daily and Three Times Weekly in Patients With Dialysis-Dependent CKD With Anemia
Source: Kidney360. 2024 Sep 4;5(11):1652–61. doi: 10.34067/KID.0000000567 (PMC12282625; doi:10.34067/KID.0000000567)
Supplement: SUPPLEMENTARY MATERIAL [file kidney360-5-1652-s001.pdf]

ASN American Society of Nephrology | JASN Journal of the American Society of Nephrology | KIDNEY360 Accessing Our World From Every Angle | CJASN Clinical Journal of the American Society of Nephrology

ASN Journal Disclosure Form

As per ASN journal policy, I have disclosed any financial relationships or commitments I have held in the past 36 months as included below. I have listed my Current Employer below to indicate there is a relationship requiring disclosure. If no relationship exists, my Current Employer is not listed.

S. Burke reports the following:
Employer: Akebia Therapeutics; Consultancy: Endomimetics; Ownership Interest: Akebia Therapeutics; Protara Therapeutics; Patents or Royalties: Pharmacosmos; and Other Interests or Relationships: American Kidney Fund fundraising event annually.

I understand that the information above will be published within the journal article, if accepted, and that failure to comply and/or to accurately and completely report the potential financial conflicts of interest could lead to the following: 1) Prior to publication, article rejection, or 2) Post-publication, sanctions ranging from, but not limited to, issuing a correction, reporting the inaccurate information to the authors' institution, banning authors from submitting work to ASN journals for varying lengths of time, and/or retraction of the published work.

Name: Steven K. Burke
Manuscript ID: K360-2024-000137R1
Manuscript Title: Safety and Efficacy of Vadadustat Once-Daily and 3-Times-Weekly in Dialysis-Dependent Chronic Kidney Disease Patients With Anemia.
Date of Completion: July 1, 2024
Disclosure Updated Date: May 14, 2024

ASN American Society of Nephrology | JASN Journal of the American Society of Nephrology | KIDNEY360 Accessing Our World From Every Angle | CJASN Clinical Journal of the American Society of Nephrology

ASN Journal Disclosure Form

As per ASN journal policy, I have disclosed any financial relationships or commitments I have held in the past 36 months as included below. I have listed my Current Employer below to indicate there is a relationship requiring disclosure. If no relationship exists, my Current Employer is not listed.

G. Hernandez reports the following:
Employer: El Paso Kidney Specialists, PA; Consultancy: Akebia; Alexion; Ardelyx; Astra Zeneca; Aurinia; Bayer; CareDx; Calliditas; Chinook; Kezar; Novartis; Otsuka; Travere; Tricida; VBI vacines; Vera Therapeutics; Vifor; Ownership Interest: DaVita Kidney Care; Research Funding: Akebia; Alexion; Apellis; Ardelyx; Astra Zeneca; Aurinia; Bayer; Boehringer Ingelheim; Bristol Myers Squibb; Cara therapeutics; Chinook; CSL Behring; Dimerix; Dynavax; Galderma; Gilead Sciences; Glaxo Smith Klein; Goldfinch Bio; IgM Biosciences; Janssen; KBP Biosciences; Kezar Life Sciences; Morphosys AG; Novartis; NovoNordisk; Otsuka; Renibus; Travere; Vera Therapeutics; Vertex; Vifor; Honoraria: Akebia; Alexion; Astra Zeneca; Aurinia; Bayer; CareDx; Calliditas; Kezar; Otsuka; Travere Therapeutics; Tricida; VBI vacines; Vifor; and Speakers Bureau: Akebia; Alexion; Astra Zeneca; Aurinia; Bayer; Boehringer Ingelheim; Calliditas; Eli Lily; Fresenius; Glaxo Smith Klein; Otsuka; Travere therapeutics; Vifor.

I understand that the information above will be published within the journal article, if accepted, and that failure to comply and/or to accurately and completely report the potential financial conflicts of interest could lead to the following: 1) Prior to publication, article rejection, or 2) Post-publication, sanctions ranging from, but not limited to, issuing a correction, reporting the inaccurate information to the authors' institution, banning authors from submitting work to ASN journals for varying lengths of time, and/or retraction of the published work.

Name: German T. Hernandez
Manuscript ID: K360-2024-000137R1
Manuscript Title: Safety and Efficacy of Vadadustat Once-Daily and 3-Times-Weekly in Dialysis-Dependent Chronic Kidney Disease Patients With Anemia
Date of Completion: August 9, 2024
Disclosure Updated Date: August 9, 2024

ASN American Society of Nephrology JASN Journal of the American Society of Nephrology KIDNEY360 Accessing Our World From Every Angle CJASN Clinical Journal of the American Society of Nephrology

ASN Journal Disclosure Form

As per ASN journal policy, I have disclosed any financial relationships or commitments I have held in the past 36 months as included below. I have listed my Current Employer below to indicate there is a relationship requiring disclosure. If no relationship exists, my Current Employer is not listed.

A. Kathresal reports the following:
Employer: Durham Nephrology Associates; and Research Funding: Principal Investigator for Clinical Trials sponsored by the following companies: CinCor, AstraZeneca, Cara, Sanifit, Bering, Alnylam, Vertex, Merck, and Pathalys,.

I understand that the information above will be published within the journal article, if accepted, and that failure to comply and/or to accurately and completely report the potential financial conflicts of interest could lead to the following: 1) Prior to publication, article rejection, or 2) Post-publication, sanctions ranging from, but not limited to, issuing a correction, reporting the inaccurate information to the authors' institution, banning authors from submitting work to ASN journals for varying lengths of time, and/or retraction of the published work.

Name: Amarnath A. Kathresal
Manuscript ID: K360-2024-000137R1)
Manuscript Title: Safety and Efficacy of Vadadustat Once-Daily and 3-Times-Weekly in Dialysis-Dependent Chronic Kidney Disease Patients With Anemia
Date of Completion: August 22, 2024
Disclosure Updated Date: August 22, 2024

ASN
American Society of Nephrology
JASN
Journal of the American Society of Nephrology
KIDNEY360
Accessing Our World From Every Angle
CJASN
Clinical Journal of the American Society of Nephrology

ASN Journal Disclosure Form

As per ASN journal policy, I have disclosed any financial relationships or commitments I have held in the past 36 months as included below. I have listed my Current Employer below to indicate there is a relationship requiring disclosure. If no relationship exists, my Current Employer is not listed.

L. Kooienga reports the following:
Employer: Colorado Kidney Care; Research Funding: Akebia Therapeutics; Alnylam Therapeutics, Ardelyx ; AstraZeneca; Boehringer Ingelheim; Cara Therapeutics; Chinook Therapeutics; CSL Behring, FibroGen Inc. ; GlaxoSmithKline Pharmaceuticals; Goldfinch Bio; Mineralys Therapeutics; Omeros; Otsuka Pharmaceutical; Reata Pharmaceuticals ; Travere Therapeutics ; Tricida Inc.; Vera Therapeutics; Visterra; Walden Therapeutics; and Advisory or Leadership Role: Otsuka US IgAN Advisory Board;.

I understand that the information above will be published within the journal article, if accepted, and that failure to comply and/or to accurately and completely report the potential financial conflicts of interest could lead to the following: 1) Prior to publication, article rejection, or 2) Post-publication, sanctions ranging from, but not limited to, issuing a correction, reporting the inaccurate information to the authors' institution, banning authors from submitting work to ASN journals for varying lengths of time, and/or retraction of the published work.

Name: Laura Kooienga
Manuscript ID: K360-2024-000137R1
Manuscript Title: Safety and Efficacy of Vadadustat Once-Daily and 3-Times-Weekly in Dialysis-Dependent Chronic Kidney Disease Patients with Anemia
Date of Completion: July 2, 2024
Disclosure Updated Date: May 8, 2024

ASN American Society of Nephrology
JASN Journal of the American Society of Nephrology
KIDNEY360 Accessing Our World From Every Angle
CJASN Clinical Journal of the American Society of Nephrology

ASN Journal Disclosure Form

As per ASN journal policy, I have disclosed any financial relationships or commitments I have held in the past 36 months as included below. I have listed my Current Employer below to indicate there is a relationship requiring disclosure. If no relationship exists, my Current Employer is not listed.

W. Luo reports the following:
Employer: Akebia Therapeutics, Inc.;; and Ownership Interest: Akebia Therapeutics, Inc.;.

I understand that the information above will be published within the journal article, if accepted, and that failure to comply and/or to accurately and completely report the potential financial conflicts of interest could lead to the following: 1) Prior to publication, article rejection, or 2) Post-publication, sanctions ranging from, but not limited to, issuing a correction, reporting the inaccurate information to the authors' institution, banning authors from submitting work to ASN journals for varying lengths of time, and/or retraction of the published work.

Name: Wenli Luo
Manuscript ID: K360-2024-000137R1
Manuscript Title: Safety and Efficacy of Vadadustat Once-Daily and 3-Times-Weekly in Dialysis-Dependent Chronic Kidney Disease Patients With Anemia
Date of Completion: July 26, 2024
Disclosure Updated Date: May 14, 2024

ASN
American Society of Nephrology
JASN
Journal of the American Society of Nephrology
KIDNEY360
Accessing Our World From Every Angle
CJASN
Clinical Journal of the American Society of Nephrology

ASN Journal Disclosure Form

As per ASN journal policy, I have disclosed any financial relationships or commitments I have held in the past 36 months as included below. I have listed my Current Employer below to indicate there is a relationship requiring disclosure. If no relationship exists, my Current Employer is not listed.

Z. Yang reports the following:
Employer: Akebia, Inc.

I understand that the information above will be published within the journal article, if accepted, and that failure to comply and/or to accurately and completely report the potential financial conflicts of interest could lead to the following: 1) Prior to publication, article rejection, or 2) Post-publication, sanctions ranging from, but not limited to, issuing a correction, reporting the inaccurate information to the authors' institution, banning authors from submitting work to ASN journals for varying lengths of time, and/or retraction of the published work.

Name: Zhihui (Sunny) Yang
Manuscript ID: k360-2024-000137R1
Manuscript Title: safty and efficacy of vadadustat once-daily and 3-times-weekly in dialysis-dependent chronic kidney disease patients with anemia
Date of Completion: July 2, 2024
Disclosure Updated Date: July 2, 2024

ASN
American Society of Nephrology
JASN
Journal of the American Society of Nephrology
KIDNEY360
Accessing Our World From Every Angle
CJASN
Clinical Journal of the American Society of Nephrology

ASN Journal Disclosure Form

As per ASN journal policy, I have disclosed any financial relationships or commitments I have held in the past 36 months as included below. I have listed my Current Employer below to indicate there is a relationship requiring disclosure. If no relationship exists, my Current Employer is not listed.

Z. Zhang reports the following:
Employer: Astrazeneca; and Ownership Interest: Akebia therapeutics.

I understand that the information above will be published within the journal article, if accepted, and that failure to comply and/or to accurately and completely report the potential financial conflicts of interest could lead to the following: 1) Prior to publication, article rejection, or 2) Post-publication, sanctions ranging from, but not limited to, issuing a correction, reporting the inaccurate information to the authors' institution, banning authors from submitting work to ASN journals for varying lengths of time, and/or retraction of the published work.

Name: Zhiqun Zhang
Manuscript ID: K360-2024-000137R1
Manuscript Title: Safety and Efficacy of Vadadustat Once-Daily and 3-Times-Weekly in Dialysis-Dependent Chronic Kidney Disease Patients With Anemia
Date of Completion: August 21, 2024
Disclosure Updated Date: August 21, 2024

ASN American Society of Nephrology
JASN Journal of the American Society of Nephrology
KIDNEY360 Accessing Our World From Every Angle
CJASN Clinical Journal of the American Society of Nephrology

ASN Journal Disclosure Form

As per ASN journal policy, I have disclosed any financial relationships or commitments I have held in the past 36 months as included below. I have listed my Current Employer below to indicate there is a relationship requiring disclosure. If no relationship exists, my Current Employer is not listed.

R. Zwiech reports the following:
Employer: Barlicki Memorial Teaching Hospital No 1 of the Medical University of Lodz; and Research Funding: salary for PI (one site) in the Vadadustat Study.

I understand that the information above will be published within the journal article, if accepted, and that failure to comply and/or to accurately and completely report the potential financial conflicts of interest could lead to the following: 1) Prior to publication, article rejection, or 2) Post-publication, sanctions ranging from, but not limited to, issuing a correction, reporting the inaccurate information to the authors' institution, banning authors from submitting work to ASN journals for varying lengths of time, and/or retraction of the published work.

Name: Rafal Zwiech
Manuscript ID: K360-2024-000137R1
Manuscript Title: Safety and Efficacy of Vadadustat Once-Daily and 3-Times-Weekly in Dialysis-Dependent Chronic Kidney Disease Patients With Anemia
Date of Completion: July 2, 2024
Disclosure Updated Date: July 2, 2024
