# Supplementary material for: Safety and Efficacy of Vadadustat Once Daily and Three Times Weekly in Patients With Dialysis-Dependent CKD With Anemia
Source: Kidney360. 2024 Sep 4;5(11):1652–61. doi: 10.34067/KID.0000000567 (PMC12282625; doi:10.34067/KID.0000000567)
Supplement: SUPPLEMENTARY MATERIAL [file kidney360-5-1652-s002.pdf]

Supplemental Appendix

Contents

	Page no.
**Supplemental Figures**	
Supplemental Figure 1. CONSORT flow chart for the MO_2DIFY trial	2
Supplemental Figure 2. Box and whisker plot of change in hemoglobin over time (randomized population)	3
Supplemental Figure 3. Mean hemoglobin values and difference between treatments for change in hemoglobin from baseline to PEP and SEP (randomized and per-protocol populations)	4
**Supplemental Tables**	
Supplemental Table 1. Study inclusion and exclusion criteria	5
Supplemental Table 2. Trial endpoints	7
Supplemental Table 3. Selected demographic baseline characteristics (per-protocol population)	8
Supplemental Table 4. Proportion of patients with average hemoglobin values within target range (randomized population)	10
Supplemental Table 5. Change in hemoglobin from baseline during primary and secondary evaluation periods by geographic region (randomized populations)	11
Supplemental Table 6. Change in hemoglobin from baseline during primary and secondary evaluation periods by baseline vadadustat dose group (randomized populations	12
Supplemental Table 7. Average weekly dose of study treatment (safety population)	13
Supplemental Table 8. Proportion of patients with any ESA rescue medications (randomized population)	14
Supplemental Table 9. Proportion of patients receiving any red blood cell transfusions (randomized population)	15
Supplemental Table 10. Abnormal lab results related to liver enzymes (safety population)	16
Supplemental Table 11. Adverse events of special interest (safety population)	17
Supplemental Table 12. Hemoglobin-related safety endpoints (safety population)	18
Supplemental Table 13. Change in iron parameters over the treatment period (safety population)	19
**Supplemental Methods**	21

Supplemental Figure 1. CONSORT flow chart for the MO$_2$DIFY trial

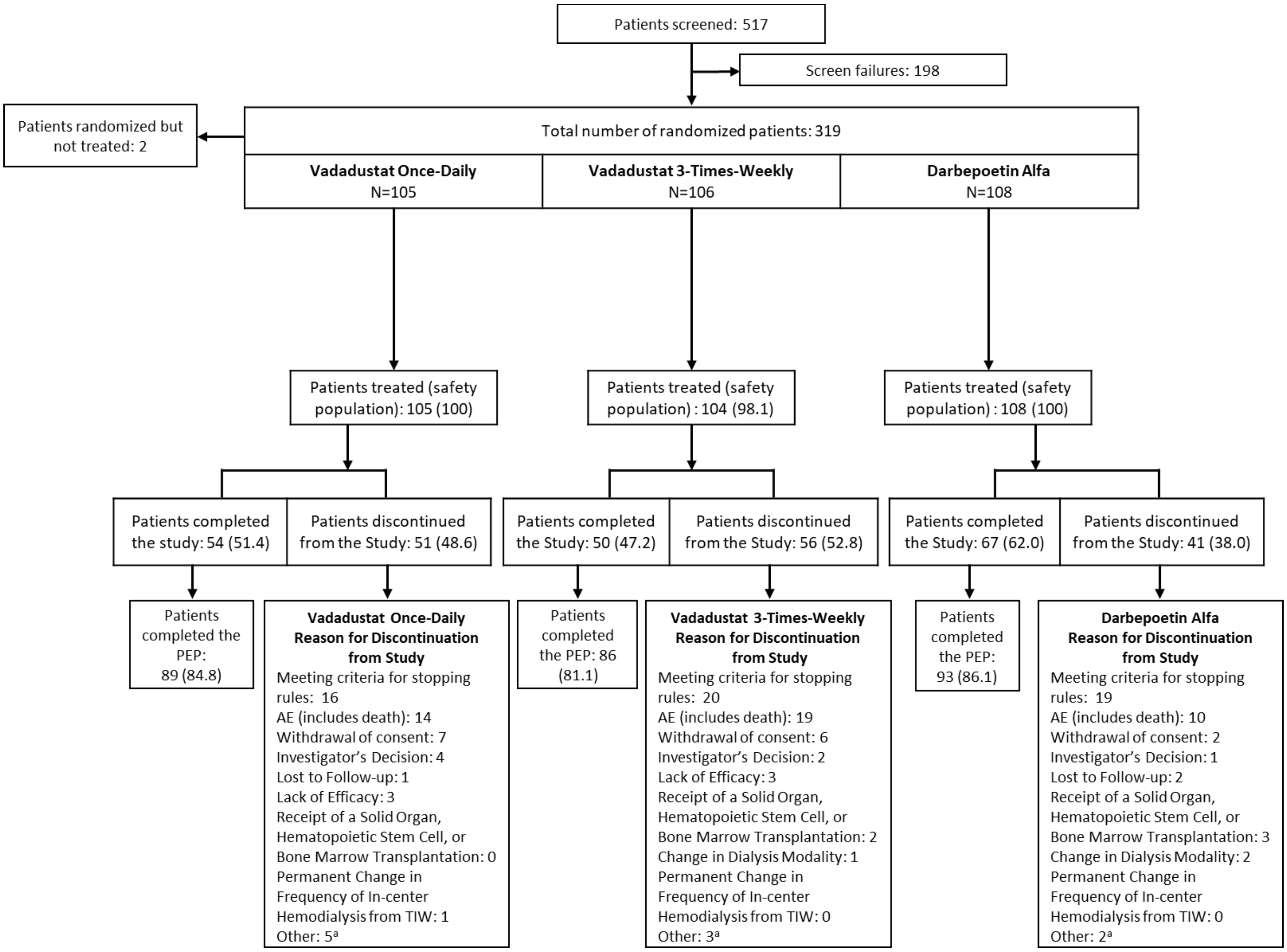

Note that the study drug was permanently discontinued if a patient met 1 of the following criteria: the first cycle of ESA rescue lasted >30 days or the patient required a second cycle of ESA for rescue (that must be related to anemia) at any time; the patient had ALT or AST >3 × ULN and total bilirubin >2 × ULN; the patient had ALT or AST >3 × ULN and international normalized ratio >1.5; ALT or AST >8 × ULN; the patient had ALT or AST remained >5 × ULN over 2 weeks (rechallenge was generally avoided with ALT or AST >5 × ULN unless there were no other good therapeutic options); the patient had ALT or AST >3 × ULN with symptoms (eg, fatigue, nausea, vomiting, right upper quadrant pain, fever, and rash) or eosinophilia.

[a]Other reasons: 8 patients had transfer of dialysis unit, 1 patient was randomized in error, and 1 patient was released from the study by the sponsor.

AE, adverse event; ALT, alanine aminotransferase; AST, aspartate aminotransferase; ESA, erythropoiesis-stimulating agent; PEP, primary evaluation period; ULN, upper limit of normal.

Supplemental Figure 2. Box and whisker plot of change in hemoglobin over time (randomized population)

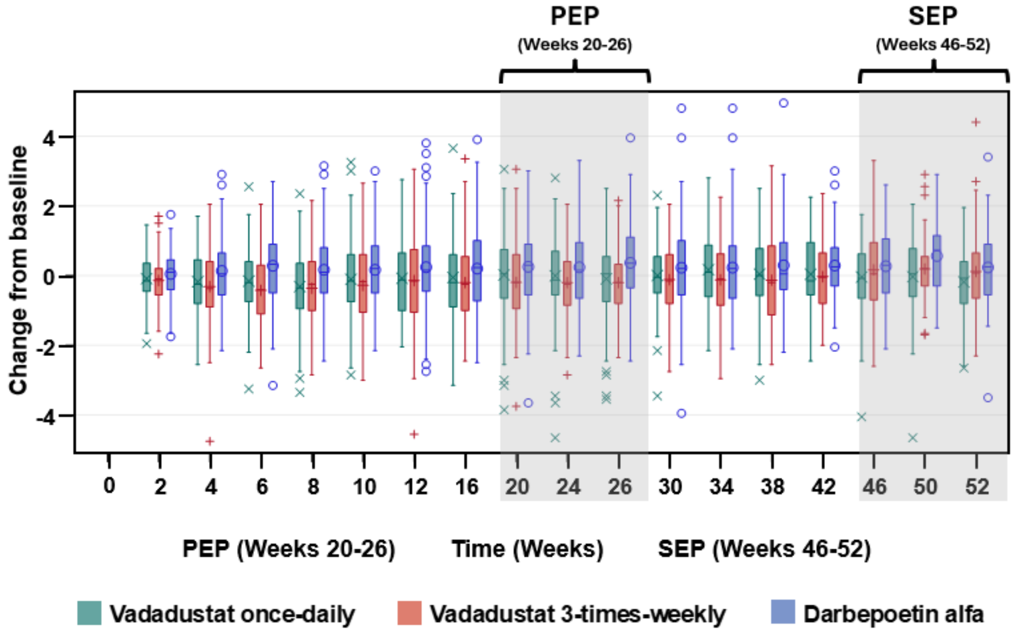

PEP, primary evaluation period; SEP, secondary evaluation period.

Supplemental Figure 3. Mean hemoglobin values and difference between treatments for change in hemoglobin from baseline to PEP and SEP (randomized and per-protocol populations)

	Vadadustat	Darbepoetin alfa	Treatment difference (Vadadustat - darbepoetin alfa)	Difference in Hb change from darbepoetin alfa, LS mean (95% CI)
**Randomized population**				
**Vadadustat Once-Daily**				
**Primary Evaluation Period (Weeks 20-26)**				
Hb concentration (observed + imputed), g/dl, mean (SD)	10.22 (1.13)	10.47 (0.91)		–0.27 (–0.55 to 0.01)
**Secondary Evaluation Period (Weeks 46-52)**				
Hb concentration (observed + imputed), g/dl, mean (SD)	10.20 (1.00)	10.59 (1.16)		–0.40 (–0.79 to –0.02)
**Vadadustat Three-Times-Weekly**				
**Primary Evaluation Period (Weeks 20-26)**				
Hb concentration (observed + imputed), g/dl, mean (SD)	9.94 (0.96)	10.47 (0.91)		–0.53 (–0.80 to –0.25)
**Secondary Evaluation Period (Weeks 46-52)**				
Hb concentration (observed + imputed), g/dl, mean (SD)	10.17 (1.32)	10.59 (1.16)		–0.42 (–0.81 to –0.02)
**Per protocol population**				
**Vadadustat Once-Daily**				
**Primary Evaluation Period (Weeks 20-26)**				
Hb concentration (observed + imputed), g/dl, mean (SD)	10.35 (1.01)	10.50 (0.81)		–0.09 (–0.38 to 0.21)
**Secondary Evaluation Period (Weeks 46-52)**				
Hb concentration (observed + imputed), g/dl, mean (SD)	10.31 (0.88)	10.72 (1.01)		–0.37 (–0.76 to 0.01)
**Vadadustat Three-Times-Weekly**				
**Primary Evaluation Period (Weeks 20-26)**				
Hb concentration (observed + imputed), g/dl, mean (SD)	10.05 (0.89)	10.50 (0.81)		–0.44 (–0.73 to –0.16)
**Secondary Evaluation Period (Weeks 46-52)**				
Hb concentration (observed + imputed), g/dl, mean (SD)	10.36 (1.28)	10.72 (1.01)		–0.36 (–0.75 to 0.02)

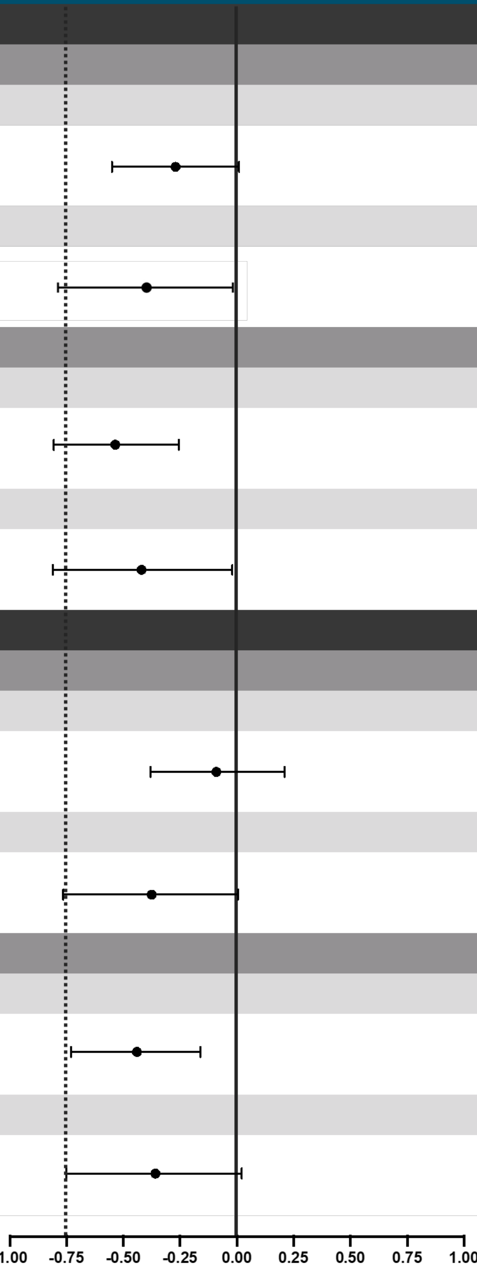

The noninferiority of vadadustat once-daily to darbepoetin alfa and vadadustat 3-times-weekly was defined as above the prespecified noninferiority margin of –0.75 g/dL.
Hb, hemoglobin; LS, least squares.

Supplemental Table 1. Study inclusion and exclusion criteria

**Inclusion criteria**
≥18 years of age
Receiving chronic outpatient 3-times-weekly in-center hemodialysis for ESRD for at least 12 weeks prior to Screening
Hemodialysis adequacy as indicated by single-pool Kt/Vurea ≥1.2 using the most recent historical measurement within 8 weeks prior to or during Screening
Use of any approved ESA for at least the 8 weeks prior to Screening Visit 2
Two Hb values, at least 4 days apart, measured by the central laboratory during Screening within the following prespecified ranges:<br>a) Hb values between 8.0 and 11.0 g/dL (inclusive) in the US<br>b) Hb values between 9.0 and 12.0 g/dL (inclusive) in Europe
Serum ferritin ≥100 ng/mL and TSAT ≥20% during Screening
Folate and vitamin B12 measurements ≥ lower limit of normal during Screening
**Exclusion criteria**
Women of childbearing potential who did not agree to practice 2 different methods of birth control or remained abstinent during the study and for 30 days after the last dose of IMP. If employing birth control, 2 of the following precautions must have been used: vasectomy of partner, tubal ligation, vaginal diaphragm, intrauterine device, or birth control
Male patients who have not had a vasectomy and did not agree to use an acceptable form of contraception during the study (refer to Appendix 16.1.1 Protocol Section 10.3) and for 30 days after the last dose of the study drug, and to not donate semen during the study and for at least 30 days after the last dose of vadadustat
Women who were breastfeeding and/or who had a positive pregnancy test result prior to receiving IMP
Patients with contraindication to required study assessment
Patients who, in opinion of the investigator or medical monitor, had a medical history or medical findings inconsistent with safety or study compliance
Anemia due to a cause other than CKD (eg, sickle cell disease, myelodysplastic syndromes, bone marrow fibrosis, hematologic malignancy, myeloma, hemolytic anemia, thalassemia, or pure red cell aplasia)
Patients meeting cutoff of the following equivalent mean weekly doses calculated over 8 weeks prior to Screening Visit 2:<br>a) Methoxy polyethylene glycol-epoetin beta >50 µg/week<br>b) Darbepoetin alfa >100 µg/week<br>c) Epoetin analogs >23000 IU/week
Active bleeding or recent blood loss within 8 weeks prior to randomization
RBC within 8 weeks prior to randomization
Anticipated to discontinue hemodialysis during the study
Judged by the investigator that the patient was likely to need rescue therapy (ESA administration or RBC transfusion) immediately after enrollment in the study
History of chronic liver disease (eg, chronic infectious hepatitis, chronic autoimmune liver disease, cirrhosis, or fibrosis of the liver)
AST/SGOT, ALT/ SGPT, or total bilirubin >1.5 Å~ ULN during Screening. Patients with a history of Gilbert's syndrome were not excluded
Current uncontrolled hypertension as determined by the investigator that contraindicated the use of an ESA
Acute coronary syndrome (hospitalization for unstable angina or myocardial infarction), surgical or percutaneous intervention for coronary, cerebrovascular or peripheral artery disease (aortic or lower

extremity), surgical or percutaneous valvular replacement or repair, sustained ventricular tachycardia, hospitalization for HF or New York Heart Association Class IV HF, or stroke within 12 weeks prior to or during Screening
History of new or recurrent malignancy within 2 years prior to and during Screening or currently receiving treatment or suppressive therapy for cancer. Patients with treated basal cell carcinoma of skin, curatively resected squamous cell carcinoma of skin, or cervical carcinoma in situ were not excluded
History of a new or recurrent episode of deep vein thrombosis or pulmonary embolism within 12 weeks prior to or during Screening
History of hemosiderosis or hemochromatosis
History of prior organ transplantation (patients with a history of failed kidney transplant or corneal transplants were not excluded)
Scheduled organ transplant from a living donor and patients on the kidney transplant wait-list who were expected to receive a transplant within 6 months
History of a prior hematopoietic stem cell or bone marrow transplant (stem cell therapy for knee arthritis was not excluded)
Known hypersensitivity to vadadustat, darbepoetin alfa, or any of their excipients
Used an investigational medication within 30 days or 5 half-lives of the investigational medication (whichever is longer), prior to screening or during screening and any prior use of a HIF-PH inhibitor. Patients could participate in another concurrent study only if that study was a noninterventional, observational investigation
Patients with bilateral native nephrectomy
Treated with probenecid within the 28-day Screening period prior to randomization or during the study treatment duration
Any other reason, which in the opinion of the investigator, made the patients not suitable for participation in the study

ALT, alanine aminotransferase; AST, Aspartate aminotransferase; CKD, chronic kidney disease; ESA erythropoiesis-stimulating agent; ESRD, end-stage renal disease; Hb, hemoglobin; HF, heart failure; HIF-PH, hypoxia-inducible factor prolyl-hydroxylases; IMP, investigational medicinal product; RBC red blood cell; SGOT, serum glutamic oxaloacetic transaminase; SGPT, serum glutamic pyruvic transaminase; TSAT, transferrin saturation; ULN, upper limit of normal.

Supplemental Table 2. Trial endpoints

Endpoints	Measures
Primary efficacy	• Assess the change in average Hb between baseline (average pretreatment Hb) and the PEP (average Hb from weeks 20 to 26, inclusive)
Secondary efficacy	• Assess the change in Hb value between baseline (average pretreatment Hb) and the SEP (average Hb from weeks 46 to 52, inclusive)
Other efficacy	• Proportion of patients having average Hb values within the target range during the primary evaluation period (weeks 20 to 26)<br>• Proportion of patients having average Hb values within the target range during the secondary evaluation period (weeks 46 to 52)<br>• Proportion of patients with any ESA rescue medications during the PEP (weeks 20 to 26)<br>• Proportion of patients with any ESA rescue medications during the PEP (weeks 46 to 52)<br>• Proportion of patients receiving RBC transfusions from baseline to week 26<br>• Proportion of patients receiving RBC transfusions from baseline to week 52
Safety	• AEs and SAEs<br>• TEAEs and treatment-emergent SAEs<br>• AESIs<br>• Clinical laboratory values<br>• Hb excursions >11.0 g/dL for US, >12.0 g/dL, >13.0 g/dL, >14.0 g/dL, <8.0 g/dL, or <9.0 g/dL<br>• Number of episodes of Hb increase >1.0 g/dL within any 2-week interval or >2.0 g/dL within any 4-week interval

AE, adverse event; AESIs, adverse events of special interest; ESA, erythropoiesis-stimulating agent; Hb, hemoglobin; PEP, primary evaluation period; RBC red blood cell; SAEs, serious adverse events; SEP, secondary evaluation period; TEAEs, treatment-emergent AEs.

Supplemental Table 3. Selected demographic baseline characteristics (per-protocol population)

Characteristic	Vadadustat Once-Daily (*n*=62)	Vadadustat 3-Times-Weekly (*n*=64)	Darbepoetin Alfa (*n*=64)
Mean age, years (SD)	60.7 (13.3)	62.4 (11.5)	58.9 (13.2)
Sex, male, *n* (%)	32 (51.6)	40 (62.5)	42 (65.6)
Racial or ethnic group, *n* (%)			
White	37 (59.7)	44 (68.8)	46 (71.9)
Black	20 (32.3)	17 (26.6)	17 (26.6)
Asian	3 (4.8)	0 (0.0)	1 (1.6)
Hispanic ethnic group, *n* (%)			
Hispanic/Latino	13 (21.0)	20 (31.3)	14 (21.9)
Not Hispanic/Latino	49 (79.0)	44 (68.8)	50 (78.1)
Region of enrollment, *n* (%)			
United States	47 (75.8)	41 (64.1)	40 (62.5)
Europe	15 (24.2)	23 (35.9)	24 (37.5)
Dry weight, kg, mean (SD)	84.8 (21.4)	83.3 (21.5)	86.3 (20.5)
Mean BMI, kg/m^2 (SD)	29.9 (6.8)	29.3 (6.8)	29.8 (6.2)
Baseline ESA use, *n* (%)			
Epoetin alfa	28 (45.2)	29 (45.3)	28 (43.8)
Darbepoetin alfa	8 (12.9)	11 (17.2)	21 (32.8)
Methoxy polyethylene glycol-epoetin beta	26 (41.9)	24 (37.5)	15 (23.4)
Darbepoetin alfa (ESA equivalent dose), *n* (%)			
≤0.45 µg/kg/week	53 (85.5)	50 (78.1)	49 (76.6)
>0.45 and ≤1.5 µg/kg/week	9 (14.5)	14 (21.9)	15 (23.4)
Baseline ESA dose (U/kg/week)			
Mean (SD)	56.2 (51.4)	68.6 (50.4)	64.3 (45.3)
Median (Q1, Q3)	41.0 (22.0, 68.0)	54.0 (30.0, 87.0)	56.0 (32.0, 88.0)
≤90 U/kg/week	53 (85.5)	50 (78.1)	48 (75.0)
>90 and <300 U/kg/week	9 (14.5)	14 (21.9)	16 (25.0)
Baseline Hb			
Concentration, g/dL, mean (SD)	10.3 (0.69)	10.3 (0.77)	10.3 (0.68)
<10 g/dL, *n* (%)	19 (30.6)	21 (32.8)	21 (32.8)
≥10 g/dL, *n* (%)	43 (69.4)	43 (67.2)	43 (67.2)
Iron (µg/dL), mean (SD)	85.6 (37.35)	78.7 (34.34)	76.4 (34.08)
Ferritin (ng/mL), mean (SD)	767.6 (364.81)	689.0 (440.07)	605.5 (336.79)
TSAT (%), mean (SD)	40.8 (14.12)	37.8 (14.07)	37.6 (13.08)
Iron dose use, *n* (%)			
Patient not receiving any iron	18 (29.0)	19 (29.7)	14 (21.9)
Patient receiving IV iron only	38 (61.3)	39 (60.9)	44 (68.8)
IV iron (mg/week), mean (SD)	53.0 (27.2)	61.8 (51.4)	64.9 (52.4)
Years since chronic dialysis initiation, mean (SD)	3.9 (2.6)	4.1 (4.3)	3.8 (3.5)
NYHA CHF class, *n* (%)			

0 or I	53 (85.5)	51 (79.7)	49 (76.6)
II or III	5 (8.1)	6 (9.4)	11 (17.2)
Unknown	4 (6.5)	7 (10.9)	4 (6.3)
Comorbidities, *n* (%)			
Diabetes	38 (61.3)	37 (57.8)	41 (64.1)
Cardiovascular disease (coronary artery disease, myocardial infarction, stroke, and heart failure)	28 (45.2)	30 (46.9)	30 (46.9)
Other cardiovascular disease (deep venous thrombosis, arterial thrombosis, pulmonary embolism, and vascular access thrombosis)	9 (14.5)	9 (14.1)	7 (10.9)

BMI, body mass index; ESA, erythropoiesis-stimulating agent; Hb, hemoglobin; IV, intravenous; NYHA CHF, New York Heart Association congestive heart failure; TSAT, transferrin saturation.

Supplemental Table 4. Proportion of patients with average hemoglobin values within target range (randomized population)

	Vadadustat Once-Daily (*n*=105)	Vadadustat 3-Times-Weekly (*n*=106)	Darbepoetin Alfa (*n*=108)
**Primary Evaluation Period (Weeks 20-26)**			
Number of available patients (observed)	89	86	93
Proportion of patients (observed), *n* (%)[a]	48 (53.9)	48 (55.8)	53 (57.0)
Odds ratio vs darbepoetin alfa (95% CI)	0.89 (0.48 to 1.63)	0.94 (0.51 to 1.76)	–
Number of available patients (observed + imputed)	105	106	108
Proportion of patients (observed + imputed), *n* (%)[a]	54 (51.0)	54 (50.7)	59 (54.5)
Odds ratio vs darbepoetin alfa (95% CI)	0.87 (0.48 to 1.59)	0.86 (0.47 to 1.60)	–
**Secondary Evaluation Period (Weeks 46-52)**			
Number of available patients (observed)	61	60	73
Proportion of patients (observed), *n* (%)[a]	37 (60.7)	38 (63.3)	44 (60.3)
Odds ratio vs darbepoetin alfa (95% CI)	1.05 (0.50 to 2.18)	1.04 (0.49 to 2.17)	–
Number of available patients (observed + imputed)	105	106	108
Proportion of patients (observed + imputed), *n* (%)[a]	53 (50.4)	51 (48.3)	55 (51.3)
Odds ratio vs darbepoetin alfa (95% CI)	0.96 (0.49 to 1.90)	0.89 (0.46 to 1.70)	–

[a]The proportion of patients at each visit period are calculated as follows: for observed data, percentage is calculated based on number of available patients; for (observed + imputed) data, *n* (%) of responders is calculated as the average *n* (%) of responders based on 100 imputation data sets.

Supplemental Table 5. Change in hemoglobin by geographic region (randomized populations)

	Vadadustat Once-Daily	Vadadustat 3-Times-Weekly	Darbepoetin Alfa
**United States**			
**Randomized Population, *n***	75	77	77
Baseline Hb concentration, g/dL, mean (SD)	10.08 (0.57)	9.96 (0.68)	9.96 (0.76)
**PEP (Weeks 20-26)**			
Hb concentration (observed + imputed), g/dL, mean (SD)	10.03 (1.02)	9.70 (0.95)	10.30 (0.86)
Change in Hb from baseline,[a] LS mean (95% CI)	0.02 (–0.23, 0.26)	–0.29 (–0.53, –0.05)	0.31 (0.07, 0.55)
Difference in Hb change from darbepoetin alfa, LS mean (95% CI)	–0.30 (–0.63, 0.03)	–0.61 (–0.93, –0.28)	–
**SEP (Weeks 46-52)**			
Hb concentration (observed + imputed), g/dL, mean (SD)	9.96 (0.94)	9.97 (1.38)	10.36 (1.13)
Change in Hb from baseline,[a] LS mean (95% CI)	–0.09 (–0.42, 0.24)	–0.07 (–0.43, 0.30)	0.33 (–0.02, 0.68)
Difference in Hb change from darbepoetin alfa, LS mean (95% CI)	–0.42 (–0.88, 0.05)	–0.39 (–0.89, 0.10)	–
**Europe**			
**Randomized Population, *n***	30	29	31
Baseline Hb concentration, g/dL, mean (SD)	10.87 (0.70)	10.78 (0.55)	10.76 (0.62)
**PEP (Weeks 20-26)**			
Hb concentration (observed + imputed), g/dL, mean (SD)	10.68 (1.25)	10.57 (0.66)	10.88 (0.91)
Change in Hb from baseline,[a] LS mean (95% CI)	–0.29 (-0.84, 0.26)	–0.41 (–0.91, 0.09)	–0.08 (–0.56, 0.40)
Difference in Hb change from darbepoetin alfa, LS mean (95% CI)	–0.21 (–0.76, 0.35)	–0.33 (–0.83, 0.18)	–
**SEP (Weeks 46-52)**			
Hb concentration (observed + imputed), g/dL, mean (SD)	10.81 (0.86)	10.70 (0.98)	11.17 (1.01)
Change in Hb from baseline,[a] LS mean (95% CI)	–0.16 (–0.70, 0.38)	–0.27 (–0.80, 0.27)	0.22 (–0.33, 0.77)
Difference in Hb change from darbepoetin alfa, LS mean (95% CI)	–0.38 (–0.97, 0.21)	–0.48 (–1.07, 0.11)	–

[a]Derived from an analysis of covariance with randomization stratification factors and baseline Hb as covariates.
Hb, hemoglobin; LS, least squares; PEP, primary evaluation period; SEP, secondary evaluation period.

Supplemental Table 6. Change in hemoglobin by baseline vadadustat dose group (randomized populations)

	Baseline Darbepoetin Alfa Low Dose (≤0.45 ug/kg/week)			Baseline Darbepoetin Alfa High Dose (>0.45 and ≤1.5 ug/kg/week)		
	Vadadustat Once-Daily (300 mg initial dose)	Vadadustat 3-Times-Weekly (600 mg initial dose)	Darbepoetin Alfa [a]	Vadadustat Once-Daily (450 mg initial dose)	Vadadustat 3-Times-Weekly (750 mg initial dose)	Darbepoetin Alfa [a]
**Randomized Population, *n***	80	84	85	25	21	23
Baseline Hb concentration, g/dL, mean (SD)	10.38 (0.66)	10.24 (0.68)	10.20 (0.83)	10.05 (0.81)	9.97 (0.94)	10.15 (0.74)
**PEP (Weeks 20-26)**						
Hb concentration (observed + imputed), g/dL, mean (SD)	10.39 (1.03)	9.88 (1.01)	10.49 (0.91)	9.65 (1.25)	10.15 (0.70)	10.39 (0.90)
Change in Hb from baseline,[b] LS mean (95% CI)	0.18 (–0.05, 0.41)	–0.28 (–0.50, –0.06)	0.33 (0.11, 0.56)	–0.27 (–0.82, 0.29)	0.24 (–0.32, 0.80)	0.48 (–0.03, 0.99)
Difference in Hb change from darbepoetin alfa, LS mean (95% CI)	–0.15 (–0.47, 0.16)	–0.61 (-0.91, –0.32)	–	–0.75 (–1.36, –0.14)	–0.24 (–0.85, 0.37)	–
**SEP (Weeks 46-52)**						
Hb concentration (observed + imputed), g/dL, mean (SD)	10.29 (0.95)	10.24 (1.35)	10.67 (1.14)	9.93 (1.09)	9.89 (1.19)	10.32 (1.19)
Change in Hb from baseline,[b] LS mean (95% CI)	0.08 (–0.22, 0.39)	0.09 (–0.23, 0.40)	0.52 (0.21, 0.83)	0.10 (-0.54, 0.74)	0.07 (–0.66, 0.79)	0.50 (–0.13, 1.13)
Difference in Hb change from darbepoetin alfa, LS mean (95% CI)	–0.43 (-0.85, –0.01)	–0.43 (–0.87, 0.00)	–	–0.40 (–1.17, 0.37)	–0.43 (–1.25, 0.38)	–

[a]The initial dose of darbepoetin alfa was based on the current package insert for each investigational site in the US and the European Summary of Product Characteristics for all other investigational sites (non-US) for adult patients with CKD on dialysis. For patients already on darbepoetin alfa, the initial dosing regimen in the study was based on the prior dosing regimen.

[b]Derived from an analysis of covariance with randomization stratification factors and baseline Hb as covariates.

CKD, chronic kidney disease; Hb, hemoglobin; LS, least squares; PEP, primary evaluation period; SEP, secondary evaluation period.

Supplemental Table 7. Average weekly dose of study treatment (safety population)

Study Period	Vadadustat Once-Daily (*n*=105)	Vadadustat 3-Times-Weekly (*n*=106)	Darbepoetin Alfa (*n*=108)
Weeks 2-8			
N	105	104	108
Mean (SD)	2411.3 (799.40)	1928.5 (418.20)	28.9 (18.30)
Median	2383	1932	25
Q1, Q3	2100.0, 2950.0	1800.0, 2250.0	15.0, 39.5
Weeks 10-16			
N	92	94	98
Mean (SD)	2804.5 (1496.10)	2076.5 (853.70)	34.6 (31.50)
Median	2816.5	2182.5	25
Q1, Q3	1801.5, 4067.0	1440.0, 2700.0	13.0, 43.0
Weeks 20-26			
N	84	82	90
Mean (SD)	2945.1 (1807.50)	2160.2 (1091.10)	34.4 (35.50)
Median	2955	2208.5	25
Q1, Q3	1305.0, 4200.0	1311.0, 3150.0	13.0, 40.0
Weeks 30-42			
N	73	70	82
Mean (SD)	3056.6 (1662.60)	2358.4 (999.40)	37.4 (35.40)
Median	2738	2607	26
Q1, Q3	1763.0, 4200.0	1495.0, 3275.0	14.0, 45.0
Weeks 46-52			
N	58	50	69
Mean (SD)	3443.5 (1816.20)	2304.8 (934.40)	41.3 (37.40)
Median	3150	2250	30
Q1, Q3	2028.0, 4849.0	1620.0, 3150.0	19.0, 55.0

Note: For each visit, any dosing data within the analysis window are included for the summary. Day 1 is included in Weeks 2-8.

Supplemental Table 8. Proportion of patients with any ESA rescue medications (randomized population)

ESA Rescue Categories	Vadadustat Once-Daily *n* (%)	Vadadustat 3-Times-Weekly *n* (%)	Darbepoetin Alfa *n* (%)	Treatment Difference From Darbepoetin Alfa	
				Vadadustat Once-Daily Odds Ratio (95% CI)	Vadadustat 3-Times-Weekly Odds Ratio (95% CI)
**PEP (Weeks 20-26)**					
N	92	92	96		
With ≥50% increase in darbepoetin alfa treatment	7 (7.6)	9 (9.8)	15 (15.6)	0.46 (0.18-1.17)	0.58 (0.24-1.40)
With ≥100% increase in darbepoetin alfa treatment	7 (7.6)	9 (9.8)	9 (9.4)	0.81 (0.30-2.19)	1.03 (0.39-2.73)
**SEP (Weeks 46-52)**					
N	65	65	75		
With ≥50% increase in darbepoetin alfa treatment	1 (1.5)	1 (1.5)	9 (12.0)	0.10 (0.01-0.85)	0.12 (0.01-0.95)
With ≥100% increase in darbepoetin alfa treatment	1 (1.5)	1 (1.5)	4 (5.3)	0.27 (0.03-2.47)	0.28 (0.03-2.45)

For all treatment arms, rescue therapy is defined as rescue for worsening anemia (Hb <9.5 g/dL) with ESA medication or red blood cell transfusion, not starting after permanent study treatment discontinuation. For the darbepoetin alfa arm, darbepoetin alfa with increases in dose ≥50% or ≥100% will be considered as ESA rescue medication.
ESA, erythropoiesis-stimulating agent; Hb, hemoglobin; PEP, primary evaluation period; SEP, secondary evaluation period.

Supplemental Table 9. Proportion of patients receiving any red blood cell transfusions (randomized population)

RBC Transfusion Categories	Vadadustat Once-Daily	Vadadustat 3-Times-Weekly	Darbepoetin Alfa *n* (%)
**Proportion of patients with RBC transfusions**			
**Weeks 2-8, *N***	105	104	108
Patients with transfusion, *n* (%)	0	3 (2.9)	1 (0.7)
Odds ratio (95% CI)	NA	NA	–
**Weeks 10-16, *N***	99	100	105
Patients with transfusion, *n* (%)	0	0	1 (1.0)
Odds ratio (95% CI)	NA	NA	–
**Weeks 20-26, *N***	92	92	96
Patients with transfusion, *n* (%)	0	2 (2.2)	0
Odds ratio (95% CI)	NA	NA	–
**Weeks 30-42, *N***	81	80	91
Patients with transfusion, *n* (%)	1 (1.2)	0	1 (1.1)
Odds ratio (95% CI)	1.15 (0.07-19.60)	NA	–
**Weeks 46-52, *N***	89	88	111
Patients with transfusion, *n* (%)	0	0	0
Odds ratio (95% CI)	NA	NA	–
**Weeks 2-52, *N***	105	104	108
Patients with transfusion, *n* (%)	1 (1.0)	4 (3.9)	2 (1.9)
Odds ratio (95% CI)	0.51 (0.04-5.91)	2.08 (0.38-11.41)	–

For all treatment arms, rescue therapy for anemia was defined as rescue for worsening anemia (Hb <9.5 g/d) with ESA medication or RBC transfusion, not starting after permanent study treatment discontinuation. For the darbepoetin alfa arm, darbepoetin alfa with increases in dose ≥50% or ≥100% will be considered as ESA rescue medication.
ESA, erythropoiesis-stimulating agent; Hb, hemoglobin; NA, not available; RBC, red blood cell.

Supplemental Table 10. Abnormal lab results related to liver enzymes (safety population)

	**Vadadustat Once-Daily (*n*=102) *n* (%)**	**Vadadustat 3-Times-Weekly (*n*=102) *n* (%)**	**Darbepoetin Alfa (*n*=106) *n* (%)**
ALT >3´ ULN, U/L	1 (1.0)	0 (0.0)	2 (1.9)
AST >3´ ULN, U/L	1 (1.0)	0 (0.0)	0 (0.0)
Bilirubin >2´ ULN, mg/dL	0 (0.0)	0 (0.0)	2 (1.9)

ALT, alanine aminotransferase; AST, aspartate aminotransferase; ULN, upper limit of normal.

Supplemental Table 11. Adverse events of special interest (safety population)

Category	Vadadustat Once-Daily *N*=105		Vadadustat 3-Times-Weekly *N*=104		Darbepoetin Alfa *N*=108	
	n (%)	PY=86.75 E (E*100/PY)	*n* (%)	PY=86.75 E (E*100/PY)	*n* (%)	PY=96.14 E (E*100/PY)
Any AESIs	29 (27.6)	70 (80.7)	40 (38.5)	89 (103.4)	34 (31.5)	76 (79.1)
Worsening of hypertension	7 (6.7)	12 (13.8)	14 (13.5)	20 (23.2)	11 (10.2)	13 (13.5)
Hepatotoxicity	4 (3.8)	6 (6.9)	3 (2.9)	4 (4.6)	5 (4.6)	5 (5.2)
Pulmonary hypertension	6 (5.7)	9 (10.4)	4 (3.8)	5 (5.8)	3 (2.8)	4 (4.2)
Malignancies including renal cell carcinoma	1 (1.0)	1 (1.2)	3 (2.9)	3 (3.5)	4 (3.7)	4 (4.2)
Congestive heart failure	9 (8.6)	12 (13.8)	7 (6.7)	7 (8.1)	2 (1.9)	3 (3.1)
Thrombosis[a]	11 (10.5)	13 (15.0)	15 (14.4)	23 (26.7)	13 (12.0)	14 (14.6)
Device/shunt thrombosis/occlusion/ malfunction/stenosis[b]	6 (5.7)	7 (8.1)	10 (9.6)	11 (12.8)	13 (12.0)	14 (14.6)
Device/shunt thrombosis[c]	5 (4.8)	5 (5.8)	8 (7.7)	9 (10.5)	11 (10.2)	11 (11.4)
Seizure	2 (1.9)	3 (3.5)	0 (0.0)	0 (0.0)	1 (0.9)	1 (1.0)
Stroke	0 (0.0)	0 (0.0)	2 (1.9)	2 (2.3)	2 (1.9)	2 (2.1)
Sepsis/septic shock	2 (1.9)	2 (2.3)	5 (4.8)	5 (5.8)	5 (4.6)	5 (5.2)

n (%) = number (percent) of patients with an event; E = number of events; PY = sum of (last follow-up date for events – first dose date + 1)/365.25 for all patients; E*100/PY = event rate per 100 patient-years.

[a]Defined by the FDA as including cerebral infarction, embolic cerebral infarction, ischemic stroke, cerebellar infarction, lacunar stroke, embolic stroke, brain stem stroke, lacunar infarction, thrombosis in device, arteriovenous fistula thrombosis, arteriovenous graft thrombosis, vascular access site thrombosis, vascular graft thrombosis, graft thrombosis, shunt thrombosis, acute myocardial infarction, myocardial infarction, deep vein thrombosis, thrombosis, atrial thrombosis, peripheral artery thrombosis, subclavian vein thrombosis, brachiocephalic vein thrombosis, subclavian artery thrombosis, vena cava thrombosis, thrombophlebitis superficial, arterial thrombosis, thrombophlebitis, jugular vein thrombosis, venous thrombosis, pelvic venous thrombosis, venous thrombosis limb, cardiac ventricular thrombosis, and intracardiac thrombus.

[b]Defined by the FDA as including thrombosis in device, arteriovenous fistula thrombosis, arteriovenous graft thrombosis, vascular access site thrombosis, vascular graft thrombosis, medical device site thrombosis, device occlusion, arteriovenous fistula occlusion, vascular access site occlusion, vascular access complication, vascular access malfunction, arteriovenous graft site stenosis, shunt occlusion, shunt malfunction, vascular graft stenosis, anastomotic stenosis, vascular access site complication, and vascular graft occlusion.

[c]Defined by the FDA as including thrombosis in device, arteriovenous fistula thrombosis, arteriovenous graft thrombosis, vascular access site thrombosis, vascular graft thrombosis, graft thrombosis, shunt thrombosis, medical device site thrombosis, device related thrombosis, and injection site thrombosis.

AESIs, adverse events of special interest; FDA, US Food and Drug Administration; PY, patient-years.

Supplemental Table 12. Hemoglobin-related safety endpoints (safety population)

	Vadadustat Once-Daily (*n*=105)	Vadadustat 3-Times-Weekly (*n*=102)	Darbepoetin Alfa (*n*=106)
Hb >11.0 g/dL,[a] N1[b]	75	73	76
n (%)	47 (62.7)	40 (54.8)	63 (82.9)
Odds ratio (95% CI), (vada-DA)	0.33 (0.15-0.71)	0.25 (0.11-0.53)	
Hb >12.0 g/dL,[a] N1[b]	105	102	106
n (%)	25 (23.8)	15 (14.7)	42 (39.6)
Odds ratio (95% CI), (vada-DA)	0.42 (0.22-0.80)	0.24 (0.12-0.48)	
Hb >13.0 g/dL,[a] N1[b]	105	102	106
n (%)	2 (1.9)	1 (1.0)	7 (6.6)
Odds ratio (95% CI), (vada-DA)	0.27 (0.05-1.36)	0.14 (0.02-1.17)	
Hb >14.0 g/dL,[a] N1[b]	105	102	106
n (%)	0 (0.0)	1 (1.0)	2 (1.9)
Odds ratio (95% CI), (vada-DA)	NA	0.50 (0.05-5.60)	
Hb increase >1.0 g/dL within any 2-week interval, N1[b]	105	102	106
n (%)	26 (24.8)	20 (19.6)	35 (33.0)
Odds ratio (95% CI), (vada-DA)	0.67 (0.37-1.22)	0.48 (0.25-0.93)	
Hb increase >2.0 g/dL within any 4-week interval, N1[b]	105	102	106
n (%)	12 (11.4)	12 (11.8)	18 (17.0)
Odds ratio (95% CI), (vada-DA)	0.63 (0.29-1.38)	0.65 (0.29-1.45)	

[a]Lab results from US-based participants.

[b]N1 = number of patients with available postbaseline Hb for each category. % is calculated based on N1.

DA, darbepoetin alfa; Hb, hemoglobin; NA, not available; vada, vadadustat.

Supplemental Table 13. Change in iron parameters over time (safety population)

Parameter	Vadadustat Once-Daily (*n*=105)	Vadadustat 3-Times-Weekly (*n*=104)	Darbepoetin Alfa (*n*=108)
**Hepcidin, ng/mL**			
Baseline, mean (SD)	64.8 (32.13)	60.6 (32.84)	59.7 (33.03)
Weeks 12, n<br>Mean (SD)<br>Change from baseline, mean (SD)	75<br>51.4 (30.42)<br>–14.6 (32.17)	86<br>46.0 (29.99)<br>–12.6 (31.71)	93<br>46.1 (27.27)<br>–14.0 (33.76)
Weeks 52, n<br>Mean (SD)<br>Change from baseline, mean (SD)	31<br>44.4 (24.03)<br>–28.6 (37.59)	28<br>43.5 (28.77)<br>–15.6 (36.40)	41<br>47.9 (25.64)<br>–15.3 (31.64)
**Ferritin, ng/mL**			
Baseline, mean (SD)	714.80 (336.94)	720.07 (411.06)	663.90 (387.58)
Weeks 4, n<br>Mean (SD)<br>Change from baseline, mean (SD)	98<br>715.77 (392.83)<br>5.42 (349.16)	95<br>692.50 (360.32)<br>–30.03 (316.72)	103<br>624.18 (327.95)<br>–44.48 (211.05)
Weeks 24, n<br>Mean (SD)<br>Change from baseline, mean (SD)	82<br>736.35 (507.44)<br>12.83 (423.31)	78<br>639.36 (360.89)<br>–24.02 (391.78)	85<br>668.28 (530.65)<br>26.04 (506.91)
Weeks 42, n<br>Mean (SD)<br>Change from baseline, mean (SD)	64<br>690.27 (333.84)<br>–48.44 (345.14)	61<br>716.32 (581.51)<br>44.96 (562.98)	75<br>650.32 (355.85)<br>37.27 (324.95)
**Serum iron, ug/dL**			
Baseline	81.2 (36.45)	77.5 (30.74)	77.8 (32.87)
Weeks 4, n<br>Mean (SD)<br>Change from baseline, mean (SD)	99<br>87.8 (34.56)<br>5.9 (37.31)	97<br>84.1 (31.50)<br>6.1 (33.40)	104<br>66.4 (25.70)<br>–11.9 (34.10)
Weeks 24, n<br>Mean (SD)<br>Change from baseline, mean (SD)	82<br>85.5 (34.36)<br>1.9 (51.59)	77<br>75.7 (32.96)<br>–3.4 (35.92)	85<br>69.5 (31.60)<br>–10.9 (37.00)
Weeks 42, n<br>Mean (SD)<br>Change from baseline, mean (SD)	64<br>86.1 (39.85)<br>0.5 (58.24)	61<br>80.4 (34.98)<br>6.7 (35.53)	75<br>69.2 (28.73)<br>–8.9 (37.47)
**TIBC, ug/dL**			
Baseline	199.9 (33.21)	196.5 (30.59)	192.0 (32.11)
Weeks 4, n<br>Mean (SD)	98<br>224.5 (39.50)<br>24.6 (30.79)	97<br>221.0 (35.57)<br>22.8 (21.33)	104<br>191.4 (33.59)<br>–1.2 (18.35)

Change from baseline, mean (SD)			
Weeks 24, n<br>Mean (SD)<br>Change from baseline, mean (SD)	82<br>232.3 (38.64)<br>29.9 (37.94)	77<br>216.5 (37.83)<br>18.9 (31.32)	85<br>193.6 (34.24)<br>–1.0 (22.26)
Weeks 42, n<br>Mean (SD)<br>Change from baseline, mean (SD)	64<br>230.4 (40.05)<br>28.5 (36.96)	61<br>221.7 (35.90)<br>22.6 (28.74)	71<br>194.5 (33.39)<br>–3.2 (27.33)
**TSAT, %**			
Baseline	38.77 (13.91)	38.85 (13.47)	39.65 (14.03)
Weeks 4, n<br>Mean (SD)<br>Change from baseline, mean (SD)	97<br>38.99 (13.84)<br>–0.23 (15.33)	97<br>38.46 (14.44)<br>–0.68 (15.40)	103<br>34.29 (10.94)<br>–5.68 (13.16)
Weeks 24, n<br>Mean (SD)<br>Change from baseline, mean (SD)	79<br>35.40 (11.19)<br>–3.95 (17.91)	76<br>34.41 (12.59)<br>–4.79 (15.81)	84<br>35.27 (14.13)<br>–4.79 (17.51)
Weeks 42, n<br>Mean (SD)<br>Change from baseline, mean (SD)	64<br>37.00 (14.73)<br>–3.45 (21.04)	60<br>35.65 (13.19)<br>–0.39 (15.55)	70<br>34.66 (12.01)<br>–3.17 (18.40)

TIBC, total iron-binding capacity; TSAT, transferrin saturation.

Supplemental Methods

Trial Procedures

<u>Dosing information.</u> Vadadustat was provided in oral tablets with dosages of 150 mg and 450 mg. For the low erythropoiesis-stimulating agent (ESA)-dose group, the starting vadadustat doses were either 300 mg once-daily or 600 mg 3-times-weekly. The starting vadadustat dose for the high ESA-dose group was either 450 mg once-daily or 750 mg 3-times-weekly. The minimum vadadustat dose was 150 mg, and the maximum dose could be adjusted up to 900 mg once-daily or 1200 mg 3-times-weekly. Darbepoetin alfa was administered intravenously in the venous return line of the dialysis access by the staff at the site facility in accordance with local practice. The initial darbepoetin alfa dose was based on the patient's prior or current darbepoetin alfa dose or, if the patient was switching from a different ESA, on the conversion factor recommended in the local product label. Hemoglobin concentrations were monitored throughout the study to determine the need for dose adjustments. Both vadadustat and darbepoetin alfa doses were titrated using protocol-specified algorithms to maintain target hemoglobin levels (10-11 g/dL in the United States; 10-12 g/dL in Europe). Iron supplementation was recommended to sustain serum ferritin concentrations at or above 100 ng/mL and transferrin saturation at or above 20%.

<u>Vadadustat Dose Selection</u>

The initial doses of vadadustat were determined based on the previously completed trials in the vadadustat clinical program and pharmacokinetic/pharmacodynamic simulations. An initial phase 2 study (NCT02260193) evaluated the hematologic pharmacodynamic response, safety,

and tolerability of a vadadustat starting dose of 300 or 450 mg daily and 450 mg 3-times-weekly administered orally for 16 weeks in patients undergoing dialysis and previously receiving epoetin alfa. All 3 regimens maintained stable hemoglobin levels throughout the 16-week treatment period, as assessed by the primary endpoint of the change in hemoglobin to midtrial (weeks 7-8) and end-of-trial (weeks 15-16).[1] There was only 1 hemoglobin excursion >13g/dL. In this study, the ratio of total 3-times-weekly dose to total daily dose was between 1.0 to 1.67. We expected that the starting doses of 600 mg 3-times-weekly (twice the daily dose) and 750 mg 3-times-weekly (1.67 times the daily dose), along with dose titration, would achieve and maintain hemoglobin levels within the target range (10.0 to 11.0 g/dL).

Rescue for Anemia. Patients in all treatment groups were eligible to receive an ESA or red blood cell (RBC) transfusion as rescue therapy if they experienced worsening symptoms of anemia, with a hemoglobin concentration <9.5 g/dL. Rescue therapy was halted at hemoglobin levels ≥10.0 g/dL following the local institution's guidelines and the product label. Rescue therapy involved rescuing patients with hemoglobin <9.5 g/dL using ESA medication or RBC transfusion. In the darbepoetin alfa arm, ESA rescue medication was defined by increases in darbepoetin alfa dose of either ≥50% in one analysis or ≥100% in a second analysis. During rescue therapy, vadadustat dosages were temporarily discontinued while patients received an ESA. RBC transfusion was administered as clinically indicated, and trial drugs were continued during the transfusion period. During the trial, study investigators actively collected and recorded potential adverse events at each study visit.

Adverse Events of Special Interest. AESIs included worsening of hypertension, hepatotoxicity, pulmonary hypertension, malignancies including renal cell carcinoma, thrombosis, device/shunt thrombosis/occlusion/malfunction/stenosis, device/shunt thrombosis, seizure, stroke, and sepsis/septic shock.

Analysis Populations. The randomized population included all patients randomized to the treatment assignments. The full analysis set comprised all patients in the randomized population who received at least 1 dose of the study medication and had at least 1 post-dose hemoglobin assessment. The per-protocol population consisted of all randomized patients who received study medication during the primary evaluation period (weeks 20-26), had at least 1 hemoglobin assessment during this period, and did not have any critical or major protocol deviations that could affect the primary endpoint analyses. Analyses in the per-protocol population were based on the actual treatment received, with specific protocol deviation criteria applied for exclusions. A preplanned per-protocol analysis was performed as a supportive analysis to the intent-to-treat analysis. It included patients who took the study drug during the evaluation periods and had no major protocol deviations. The safety population included all patients in the randomized population who received at least 1 dose of the study medication, and analyses were based on the actual treatment received. Efficacy analyses included data from the randomized full analysis set and per-protocol populations. The safety analyses were conducted using data from the safety population.

Determination of Noninferiority. For the primary efficacy analysis, the mean change from baseline in hemoglobin for vadadustat was assumed to be the same as for the active control (darbepoetin alfa), and the common SD for the mean change from baseline was assumed to be 1.2 g/dL. Noninferiority was established based on a 2-sided 95% CI for the difference between the vadadustat groups and darbepoetin alfa group, using a noninferiority margin of –0.75 g/dL. A noninferiority margin of –0.75 g/dL was determined from a formal meta-analysis based on prior ESA trials and would preserve three-quarters of the treatment effect. Based on these assumptions and approximately 100 patients per treatment group, noninferiority tests had >90% power with consideration of a 30% dropout rate.

Data Imputation. The primary analysis used multiple imputation with analysis of covariance (ANCOVA) as the substantive model and was performed in the randomized population. Missing data were handled by treatment policy strategy. Further, missing data, including hemoglobin outcomes and any covariates used in the models, were imputed with randomized patients using multiple imputation under fully conditional specification (FCS) method. Under the assumption of FCS, data for each variable with missing values were imputed with a separate regression model allowing for all available data to be used in the imputation process. For continuous variables, the regression method was used for imputations. For categorical variables, logistic regression models were used for binary outcomes, and nominal response logistic regression for nominal responses. An ANCOVA model compared the mean change from baseline in hemoglobin between 2 treatment groups with the complete dataset from multiple imputation. The ANCOVA model contained treatment group, baseline hemoglobin level, and the 2

stratification factors (geographic region and mean weekly darbepoetin alfa dose prior to Screening Visit 2) as predictor variables. The stratification factor assignments at randomization were used in the analysis.

Sensitivity analyses on the primary analysis were repeated with imputation of data, which may have been affected by a patient having received any form of rescue (transfusion or ESA). All per-visit hemoglobin values within 4 weeks of administration rescue therapy were set as missing prior to imputation. Additionally, a mixed model for repeated measures was fit to the observed data only and was performed in the randomized population.

Reference

1. Haase VH, Chertow GM, Block GA, et al. Effects of vadadustat on hemoglobin concentrations in patients receiving hemodialysis previously treated with erythropoiesis-stimulating agents. *Nephrol Dial Transplant.* 2019;34(1):90-99.
